# Lateralised neural mechanisms facilitate auditory self-other distinction and coordination in human dyads

**DOI:** 10.1371/journal.pbio.3003935

**Published:** 2026-08-10

**Authors:** Manuel Varlet, Tomas Lenc, Sylvie Nozaradan, David Poeppel, Peter E. Keller

**Affiliations:** 1 The MARCS Institute for Brain, Behaviour and Development, Western Sydney University, Penrith, Australia; 2 School of Psychology, Western Sydney University, Penrith, Australia; 3 Institute of Neuroscience (IONS), Université catholique de Louvain (UCL), Brussels, Belgium; 4 Basque Center on Cognition, Brain and Language (BCBL), Donostia-San Sebastian, Spain; 5 International Laboratory for Brain, Music and Sound Research (BRAMS), Montreal, Quebec, Canada; 6 Department of Psychology, New York University, New York, United States of America; 7 Center for Music in the Brain, Department of Clinical Medicine, Aarhus University & The Royal Academy of Music Aarhus/Aalborg, Aarhus, Denmark; University College London, UNITED KINGDOM OF GREAT BRITAIN AND NORTHERN IRELAND

## Abstract

Coordinating actions with others is essential for successful social interaction. Such coordination, as in sports and musical performances, often relies on sound, requiring the brain to process and integrate acoustic information related to the actions of ‘self’ and ‘other’ while maintaining sufficient distinction between them. However, such self-other differentiation can be challenged during real-world auditory interactions due to self and other signals being mixed when arriving at the eardrum, creating the potential for agency confusion and impaired coordination. Here we combined dual-EEG and frequency tagging techniques to investigate the behavioural and brain mechanisms supporting the self-other distinction during a joint auditory-motor synchronisation task. Twenty-five dyads synchronised self-paced, finger pressure-controlled continuous sound modulations. The results revealed better synchronisation when participants were associated with distinct sounds (different frequency and brightness) and designated leadership roles, facilitating the processing of self and other information and their separation at both peripheral and central levels. The results also indicated that this neural facilitation was stronger in the right hemisphere than the left, as well as for self than other, suggesting critical roles of spectral information and control over one’s own actions for auditory self-other distinction and coordination. These findings highlight complementary behavioural and brain mechanisms compensating for self-other masking inherent to auditory interactions, opening new avenues to understanding interpersonal coordination and its disorders.

## Abstract 2

In order to coordinate interactions with others, people need to distinguish between self- and other-generated signals, such as sound, but how the brain maintains this separation is unclear. This study shows that distinct sounds enhance synchronization, and that right-hemisphere-dominant neural mechanisms facilitate auditory self-other distinction and coordination.

## 1. Introduction

Coordinating actions with others is critical for efficient communication, social bonding, and, ultimately, the achievement of collective goals. The human capacity for action coordination may therefore form part of the foundations upon which our societies and cultures are built [1–3]. While social interactions can take place via multiple sensory modalities, coordination through sound is often paramount, supporting a wide range of joint activities, from conversation to group music-making [4–11]. However, coordination failures—even among experts—occur frequently, including in simplified sensorimotor synchronisation laboratory tasks such as joint finger‑tapping or drumming [12–16], underscoring the substantial demands involved in establishing and maintaining precise coordination.

A central challenge when synchronising actions with others in the auditory domain is the process of maintaining the self-other distinction. Acoustic information from separate sources becomes physically mixed by the time it reaches the eardrum [17,18], creating the potential for agency confusion and impaired coordination [14,19,20]. Disentangling self‑generated from externally generated sounds can become particularly difficult when overlapping signals reach the ear and spatial cues are degraded by source proximity or reverberant acoustics.

Here, we investigate the behavioural and neural mechanisms that allow humans to compensate for such challenging acoustic conditions and optimally balance self-other integration and segregation in order to achieve precise sound‑based joint synchronisation. Across experimental paradigms and more ecological interaction settings, evidence points to two broad classes of mechanisms that support self-other differentiation and coordination in the auditory domain.

First, distinct acoustic features—including differences in fundamental frequency and broader spectral structure (which influences perceived ‘timbre’)—provide separable spectral signatures that facilitate source separation at early stages of auditory processing [21]. Such differences give rise to partially distinct patterns of cochlea stimulation and frequency-selective encoding along the auditory pathway, enabling overlapping sounds to be distinguished within the auditory scene [17,18]. These auditory cues have been suggested to play a central role in supporting self-other differentiation during auditory interaction, including in contexts requiring precise joint auditory-motor synchronisation [14,15,22].

Second, a growing body of research shows that self-other differentiation in the auditory domain also relies on selective attentional and predictive mechanisms operating at higher levels of the auditory pathway [23–28]. Top-down modulation of auditory processing has been linked to several complementary processes, including efference-copy-based attenuation of self-generated sounds, memory-based representations of self-related auditory input, and attentional biases toward behaviourally relevant sound sources [29–33]. Related mechanisms have also been observed for other-generated sounds across a range of auditory interaction scenarios, from rhythmic synchronisation to speech processing, where attention and prediction contribute to the selective enhancement of relevant acoustic streams amid competing inputs [34–37]. The internal simulation of others’ actions is thought to contribute to these effects, with forward models of observed behaviour potentially giving rise to efference‑copy–like predictions of expected sensory consequences that support anticipatory gain control and the differentiation of self‑ and other‑produced sounds [6,19,23,38].

Together, these findings suggest that distinct acoustic features and top-down attentional and predictive mechanisms may be especially important for self-other processing and joint synchronisation in natural environments characterised by temporally overlapping and less predictable sounds. Importantly, evidence suggests that the right hemisphere plays a prominent role in processing spectral and timbral sound features relevant for sound source differentiation [39,40], and also shows specialisation for self-related auditory representations and the sense of self more broadly [29,41–43]. Related work also indicates that these mechanisms could be shaped by leadership roles during auditory interaction [15,44,45]. While leader-follower roles are sometimes explicitly assigned, they frequently emerge spontaneously during auditory coordination [46–48], potentially because leadership simplifies individual and collective action planning, increases the predictability of sensory consequences, and facilitates attention allocation toward relevant sound sources. Within this framework, self-other differentiation emerges from dynamic interactions between sensory encoding and higher-level expectations about which sound source is relevant and who is initiating changes, rather than from low-level acoustics alone.

In the present study, we examined how these factors jointly contribute to humans’ ability to regulate self-other integration/segregation and achieve precisely temporally synchronised actions through auditory information. We manipulated participants’ leadership roles and fundamental sound frequencies in a novel interpersonal improvisation paradigm in which pairs of participants synchronised self-paced fluctuations in continuous auditory streams presented binaurally and generated through ongoing fine‑grained force adjustments made with the index-finger. This auditory-motor analogue of the mirror game paradigm [49] capitalises on the informativeness of imitation-based tasks for studying self-other processing [50,51], while emphasising the dynamic predictive and attentional demands that characterise everyday auditory interaction.

Crucially, we combined this paradigm with dual-EEG recordings and frequency tagging of the continuous auditory streams, enabling us to capture distinct neurophysiological markers of self- and other-related processing, as well as of their relative integration and segregation. This dual-brain tagging approach, previously applied to visually mediated interpersonal coordination [52], provides a unique opportunity to investigate the neural processes underlying behavioural coordination dynamics by disentangling sensory-related from motor-related activity—a key limitation of many current hyperscanning approaches [53–55].

Using this integrated behavioural and neural approach, we provide evidence that leadership-based, top-down selective mechanisms operate alongside low-level acoustic differences to support self-other differentiation during auditory coordination. Our results suggest that these mechanisms form a flexible processing gradient spanning peripheral and central stages of the auditory pathway, enabling effective stream segregation and thereby supporting interpersonal synchronisation.

## 2. Results

Twenty-five pairs of participants performed the joint improvisation synchronisation task while EEG was recorded. Participants were seated back-to-back and could not see each other. Each participant controlled in real-time a continuous tone via dynamic self-paced index-finger pressure modulations recorded with a force sensor. The acoustic feature of the continuous tone modulated through finger pressure was brightness, defined as the perceived clarity or sharpness of a sound and primarily determined by the spectral centroid, that is, the relative proportion of sound energy in the higher harmonics or high-frequency content [17,56]. The brightness modulation was operationalised as a linear shift from a sinusoidal waveform at minimum force to a square waveform at maximum force, with increased squareness adding upper harmonics and producing a brighter sound.

The paired participants were instructed to produce dynamic changes in sound brightness that were as original (i.e., in terms of temporal structure and magnitude) and as synchronised as possible, in line with similar “mirror game” experiments previously conducted in the visual domain [49,52]. The two auditory streams were presented together to each participant binaurally via insert earphones. Participants were given practice trials to familiarise themselves both with the mapping between finger pressure changes and corresponding changes in sound and with the synchronisation task.

**Fig 1:**
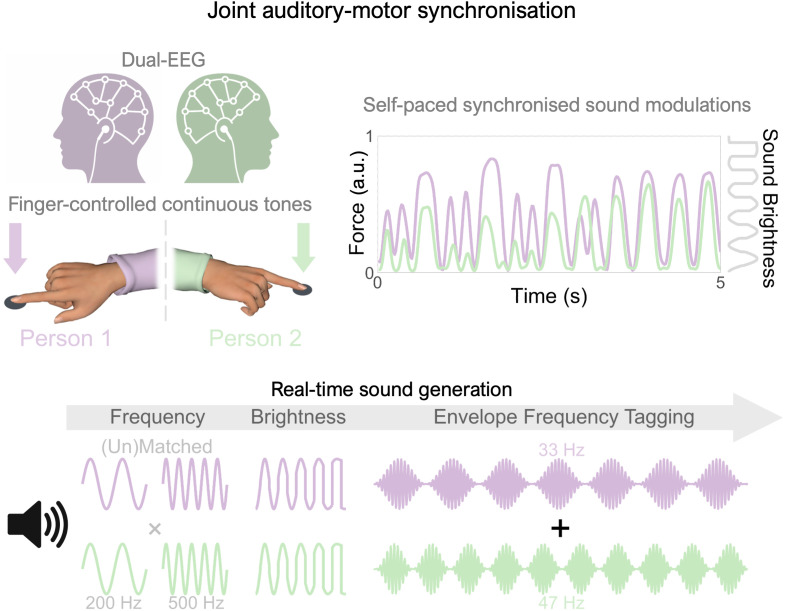
Experimental paradigm and auditory stimuli. Illustration of the improvisation synchronisation task in which paired participants produced, via dynamic finger pressure, original and synchronised fluctuations in the brightness of continuous sounds. The sound fundamental frequencies were either matched or unmatched (i.e., 200 or 500 Hz). Brightness refers to the perceptual attribute elicited by gradually changing the sound waveform from a perfect sinusoidal to a perfect square waveform, thereby adding harmonics in real-time, as a function of participants’ finger pressure. The top right panel shows a representative example of 5 s of synchronised fluctuations produced by two participants. The sound envelopes produced by Person 1 and Person 2 were tagged using a continuous sinusoidal amplitude modulation with a frequency of 33 Hz (Person 1) and 47 Hz (Person 2) before being combined and delivered binaurally to both participants (i.e., each participant received the same auditory stimuli). This frequency tagging enabled neural processing specific to self- and other-generated sounds, as well as their integration, to be indexed with high-signal-to-noise ratio across the leadership and sound frequency conditions, despite continuous changes in finger force and the resulting audio signals.

They then completed 24 3-min trials in which sound frequency and leadership roles were manipulated, resulting in a total of 72 min of recorded interaction across the experiment to ensure reliable behavioural and neural estimates. The participants’ tones had either matched or unmatched fundamental frequencies (i.e., 200 and 500 Hz), and participants were either given distinct leader and follower roles (leader-follower and follower-leader conditions) or no leadership roles (joint condition) (see Fig 1). These frequencies were selected because (i) they fall within an ecologically valid range for human vocal communication, including singing [57,58] and (ii) are low enough to elicit robust EEG responses and perceptual differences in sound brightness, while remaining clearly distinguishable [59,60].

The frequency tagging procedure consisted in tagging each participant’s sound using amplitude modulation with distinct frequencies for the self-produced and other-produced sound [61,62]. This amplitude modulation was used to elicit EEG responses specific to neural processing of the self-produced versus other-produced sound envelopes and further tag and compare these “self” and “other” EEG responses across the different leadership and sound frequency-matching conditions. Specifically, the envelope of the sounds produced by participants were tagged at 33 Hz (Person 1) and 47 Hz (Person 2) using sinusoidal amplitude modulations, as illustrated in Fig 1. In addition, neural integration of self-produced and other-produced sounds was tagged in the form of intermodulation frequencies at 14 Hz [47–33] and 80 Hz [33 + 47]. EEG responses at such intermodulation frequencies originate from nonlinear interactions between fundamental frequencies (i.e., 33 and 47 Hz) and can be used to index their integration [52,63,64], here assumed to reflect self-other integration specifically. The tagging frequencies were selected due to their proximity to 40 Hz, which is known to elicit robust auditory steady‑state EEG responses [65,66], while remaining sufficiently separated to allow clear dissociation of self‑, other‑, and intermodulation‑related signals, and high enough to minimise contamination from low‑frequency neural activity and movement‑related artefacts [67].

### 2.1. Behavioural dynamics

#### 2.1.1. Interpersonal synchronisation.

**Fig 2:**
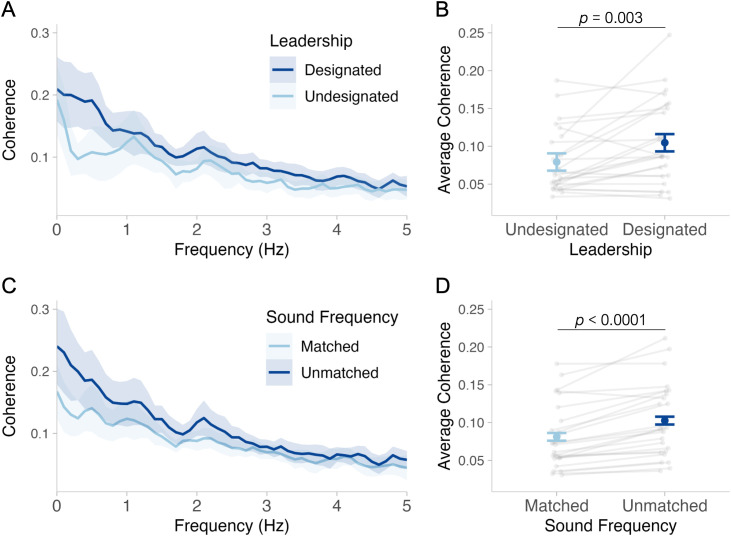
Interpersonal synchronisation. Cross-spectral coherence values indexing interpersonal synchronisation between 0 and 1 from 0 to 5 Hz (panels **A** and **C**), and averaged over this frequency range (panels **C** and **D**), as a function of the different Leadership and Sound Frequency conditions. Shaded areas and error bars represent 1 × 95% CI of the mean computed for within-subject designs [72]. Light grey lines in panels C and D represent individual pairs of participants. The data underlying this figure are reported in S1 Data (Sheet 1).

To assess the degree of synchronisation between the dynamic modulations of the two participants’ continuous tones across conditions, we calculated the cross-spectral coherence between their signals [68,69]. Coherence was computed as the normalised cross-spectrum of the two time series, obtained by dividing the magnitude of their cross-power spectral density by the product of their individual power densities. This measure captures complex alignment emerging across a range of timescales, as illustrated in Fig 2. The analysis was restricted to frequencies up to 5 Hz, encompassing the range typically associated with human rhythmic movement production and synchronisation [70,71]. Coherence values were averaged within this frequency range to obtain a single synchronisation index for subsequent analyses.

A repeated-measures ANOVA with the factors Leadership (Designated [average of leader-follower and follower-leader conditions] and Undesignated [joint condition]) and Sound Frequency (Matched and Unmatched) conducted on the average coherence values yielded significant main effects of Leadership, *F*(1, 24) = 10.67, *p* = 0.003, *η*_*g*_^*2*^ = 0.06, and Sound Frequency, *F*(1, 24) = 36.99, *p* < 0.0001, *η*_*g*_^*2*^ = 0.04. The synchronisation between the sounds produced by paired participants was stronger when leader and follower roles were designated and when the sounds had different frequencies (i.e., 200 and 500 Hz). Specifically, fluctuations in sound brightness were more highly synchronised in the designated leadership and unmatched sound frequency conditions, as indicated by higher cross-spectral coherence between the finger pressure signals producing the brightness fluctuations (see Fig 2).

This analysis did not reveal any significant interaction between Leadership and Sound Frequency, *F*(1, 24) = 2.83, *p* = 0.105, *η*_*g*_^*2*^ = 0.002 (Designated–Matched: M = 0.092, 95% CI [0.084, 0.10]; Designated–Unmatched: M = 0.118, 95% CI [0.105, 0.130]; Undesignated–Matched: M = 0.708, 95% CI [0.060, 0.082]; Undesignated–Unmatched: M = 0.088, 95% CI [0.077, 0.098]).

#### 2.1.2. Sound brightness.

**Fig 3:**
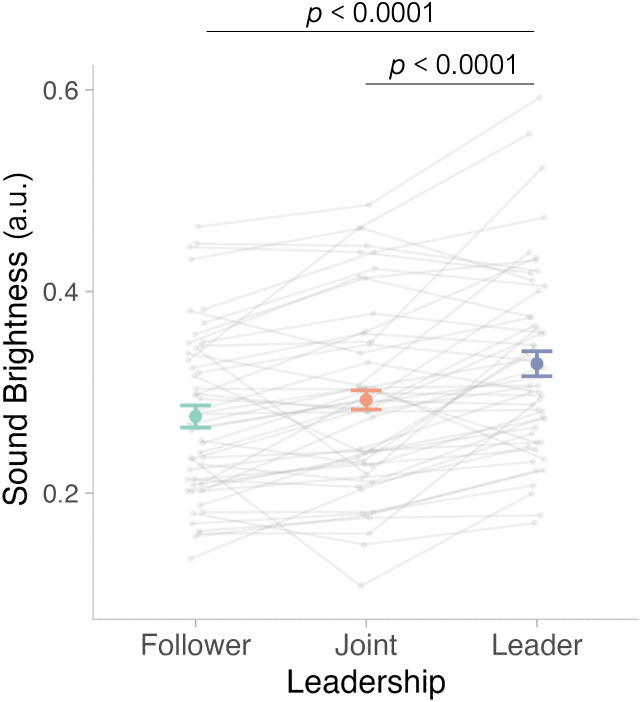
Sound brightness. Mean sound brightness (0 = perfect sinusoidal wave; 1 = perfect square wave) as a function of participant’s leadership roles. Error bars represent 1 × 95% CI of the mean computed for within-subject designs [72]. Light grey lines represent individual participants. The data underlying this figure are reported in S1 Data (Sheet 2).

Behavioural results also indicated that participants produced brighter sounds (i.e., greater finger pressure resulting in square waves with more high-amplitude harmonics) when they were instructed to lead (see Fig 3). A repeated-measures ANOVA with the factors Leadership (Leader, Follower, and Joint) and Sound Frequency (Matched and Unmatched) revealed a significant main effect of Leadership, *F*(2, 98) = 23.97, *p* < 0.0001, *η*_*g*_^*2*^ = 0.05. Pairwise comparisons (3 tests in total) with Bonferroni correction indicated that brighter sounds were produced when leading compared to when following, *t*(49) = 6.02, *p* < 0.0001, *d* = 0.59, and compared *t*o when doing the task jointly without designated leader, *t*(49) = 4.62, *p* < 0.0001, *d* = 0.39.

There was no significant main effect of Sound Frequency, *F*(1, 49) = 0.76, *p* = 0.386, *η*_*g*_^*2*^ < 0.001, with comparable values across matched (M = 0.297, 95% CI [0.285, 0.309]) and unmatched (M = 0.301, 95% CI [0.291, 0.311]) conditions. Likewise, no significant interaction between Leadership and Sound Frequency was observed, *F*(2, 98) = 0.20, *p* = 0.813, *η*_*g*_^*2*^ < 0.001 (Follower–Matched: M = 0.272, 95% CI [0.256, 0.288]; Follower–Unmatched: M = 0.280, 95% CI [0.269, 0.292]; Joint–Matched: M = 0.292, 95% CI [0.280, 0.304]; Joint–Unmatched: M = 0.293, 95% CI [0.281, 0.306]; Leader–Matched: M = 0.327, 95% CI [0.311, 0.343]; Leader–Unmatched: M = 0.330, 95% CI [0.318, 0.342]).

### 2.2. Brain dynamics

To quantify the neural tracking of self- and other-generated sounds and their integration, we performed frequency‑domain analyses on the EEG recordings. For each EEG channel, the two 180‑s trials of each of the 12 individual conditions [3 Leadership (leader-follower, follower-leader, joint) × 4 Sound frequency pairings (200−200, 500−500, 200−500, 500−200 Hz)] were first averaged, yielding a single 180‑s time series per condition. This procedure selectively enhanced the signal‑to‑noise ratio of neural responses associated with auditory tagging frequencies that were phase‑locked across trials, while attenuating unrelated neural activity [66,73]. A Fourier-based spectral analysis performed on the average trials enabled robust estimation of response amplitudes at the tagging frequencies over a total of 6 min for each condition while achieving a high spectral resolution (0.0055 Hz). This approach therefore provided a reliable measure of neural tracking of self, other, and self-other integration across extended periods of interpersonal synchronisation.

**Fig 4:**
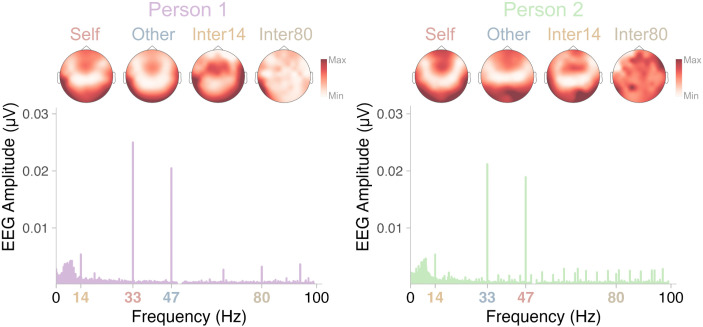
EEG frequency tagging responses and topographic distributions. EEG frequency-domain amplitude spectra (with baseline subtraction) averaged across all channels, leadership and sound conditions, and dyads (Person 1 and 2 separately). Spectra indicate significant EEG responses at Self (33 and 47 Hz for Person 1 and 2, respectively), Other (47 and 33 Hz for Person 1 and 2, respectively), and 14 and 80 Hz Intermodulation (hypothesised marker of self-other integration) frequencies evoked by the tagging of participants’ sound envelopes. The corresponding grand-averaged topographic maps (shown above the spectra), display fronto-central and occipital-temporal distributions consistent with activity originating from auditory regions (based on average re-referencing; see Materials and methods for details). The colour-scales of the topographic maps are adjusted separately for Self, Other, and Inter to their respective maximum values.

Figure 4 presents baseline-subtracted frequency-domain amplitude spectra averaged across all conditions and dyads, together with the corresponding scalp topographies. Clear responses were observed at the tagging frequencies corresponding to the participant’s own sound (Self), their partner’s sound (Other), and their integration, as reflected in significant group z-score values at 33, 47, and 14 and 80 Hz (i.e., 47–33 and 33 + 47) – see Materials and methods for further details about significance testing. The corresponding scalp distributions were consistent with auditory cortical activity, as typically observed with an average-reference montage [74,75].

Reliable responses at these frequencies were observed consistently across participants (significant z-score values for all participants for Self and Other, and 70% and 32% of the participants for 14 Hz and 80 Hz, respectively). Responses at the second harmonic of each frequency were of markedly lower amplitude and were generally less reliably present across participants (48%, 50%, 18%, and 32% of the participant sample with significant z-score values, respectively); consequently, they were not retained for further analyses. Importantly, neural responses at these frequencies *were* selectively modulated by Leadership and Sound Frequency conditions, as detailed below.

#### 2.2.1. Neural tracking of Self.

**Fig 5:**
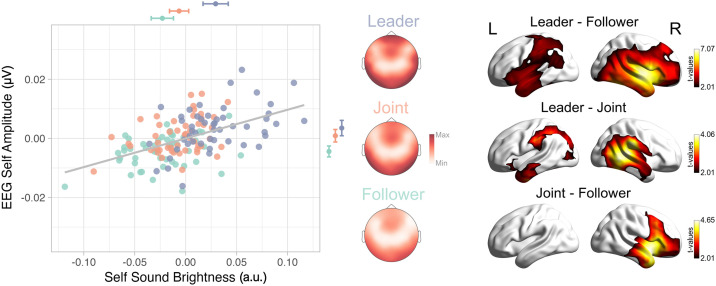
EEG Self responses as a function of sound brightness and leadership. The left panel shows the correlation between EEG Self responses and Self sound brightness, including the line of best fit. Individual participants are shown in blue, red, and green for the Leader, Joint, and Follower conditions, respectively, with the corresponding means and 95% CIs computed for within-subject designs [72] displayed on the outside. Grand-averaged topographic maps for the different leadership conditions, represented in the middle column on the same colour scale, show distributions consistent with auditory modulations. The right column shows *t*-values of significant clusters for the pairwise comparisons between the three leadership conditions in source space (based on Dynamic Imaging of Coherent Sources (DICS) and cluster-based permutation testing; see Materials and methods for details), highlighting greater involvement of the right hemisphere. The EEG data underlying this figure are reported in S1 Data (Sheet 3).

***Effect of leadership*.** A repeated-measures ANOVA on the amplitude of Self responses, with the factors Leadership (Leader, Follower, and Joint) and Sound Frequency (Matched and Unmatched), indicated a significant main effect of Leadership, *F*(2, 98) = 13.43, *p* < 0.001, *η*_*g*_^*2*^ = 0.04, as depicted in Fig 5. In line with the behavioural results for sound brightness, pairwise comparisons (3 tests in total) with Bonferroni correction indicated significantly larger Self response amplitude when leading compared to when following, *t*(49) = 4.89, *p* < 0.0001, *d* = 0.52. In*t*erestingly, and in contrast to the behavioural pattern observed for sound brightness, the comparisons also yielded significantly larger Self response amplitude when doing the task jointly (without designated leader) compared to when following, *t*(49) = 4.34, *p* < 0.001, *d* = 0.37. This dissociation sugges*t*s that the enhancement of neural responses to self-produced sounds cannot be fully explained by peripheral, stimulus-driven differences in acoustic input. Instead, it points to an additional contribution from centrally mediated mechanisms, consistent with selective enhancement of self-related auditory signals at the cortical level as a function of leadership roles.

To directly test whether leadership-related differences in Self responses could be explained by differences in sound brightness alone, we conducted multiple linear regression analyses. These analyses allowed us to statistically control for the contribution of peripheral acoustic variation while assessing the independent predictive value of leadership roles (Fig 5). This analysis showed that participants’ own sound brightness was a significant predictor of EEG Self response amplitude, *t*(146) = 4.97, *p* < 0.0001. Importantly, however, leadership roles also explained additional variance beyond sound brightness, with both Joint, *t*(146) = 3.36, *p* = 0.001, and Leader, *t*(146) = 2.73, *p* = 0.007, being significan*t* predictors. Consistent with this interpretation, a model including the factor Leadership, *F*(3, 146) = 23.85, *p* < 0.0001, *R*^*2*^ = 0.32, explained significantly more variance, *F*(2, 146) = 6.30, *p* = 0.002, than a simpler model including only Self sound brightness, *F*(1, 148) = 55.02, *p* < 0.0001, *R*^*2*^ = 0.27.

Together, these regression results further indicate that leadership-related modulations of neural responses to self-produced sounds cannot be reduced to peripheral acoustic differences alone. Rather, they suggest that participants’ interactive roles contributed independently to the cortical weighting of self-generated auditory input, supporting the interpretation of a role-dependent, top‑down modulation of self-related auditory processing.

Analyses in source space using DICS and cluster-based permutation testing, presented in Fig 5 (right column), corroborate these differences across leadership roles. These analyses also show that it is the right hemisphere that benefits the most from designated leadership roles, in line with greater sensitivity of this hemisphere to the spectral features of sounds and its involvement in self-other processing.

**Fig 6:**
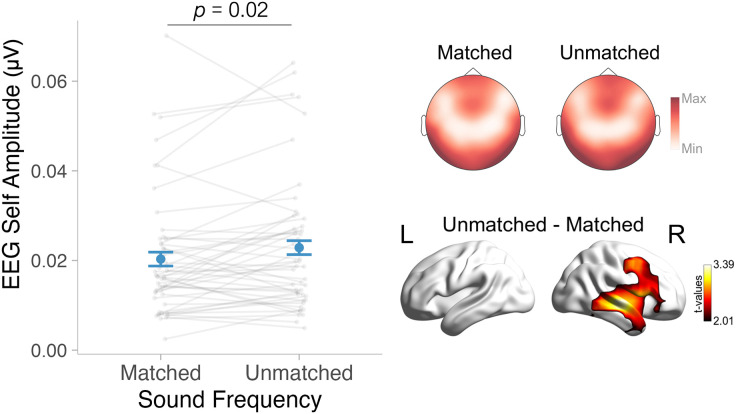
EEG Self responses for matched and unmatched sound frequency. EEG self-related responses across sound frequency conditions, with their corresponding grand-averaged topographies (presented on the same colour scale) and differences in the source space based on *t*-values of significant clusters, showing an auditory and right hemisphere advantage. Error bars represent 1 × 95% CI of the mean computed for within-subject designs [72]. Light grey lines represent individual participants. The EEG data underlying this figure are reported in S1 Data (Sheet 3).

***Effect of sound frequency*.** The ANOVA on the EEG amplitude of Self responses in the channel space also yielded a significant main effect of Sound Frequency, *F*(1, 49) = 5.48, *p* = 0.02, *η*_*g*_^*2*^ = 0.006, indicating that the neural tracking of self-produced sounds was enhanced when the frequencies of the sounds of the two participants within the pair were unmatched (see Fig 6). This result shows that the neural tracking of self-produced sounds is not only facilitated with increased sound brightness but also with distinguishable sound frequency. Source space analyses suggest that it is also the right hemisphere that benefits the most from distinguishable sound frequency for the neural tracking of Self.

No significant interaction between Leadership and Sound Frequency was observed, *F*(2, 98) = 0.21, *p* = 0.809, *η*_*g*_^*2*^ < 0.001 (Follower–Matched: M = 0.0164, 95% CI [0.0136, 0.0191]; Follower–Unmatched: M = 0.0180, 95% CI [0.0158, 0.0202]; Joint–Matched: M = 0.0213, 95% CI [0.0180, 0.0247]; Joint–Unmatched: M = 0.0237, 95% CI [0.0206, 0.0267]; Leader–Matched: M = 0.0233, 95% CI [0.0202, 0.0263]; Leader–Unmatched: M = 0.0269, 95% CI [0.0230, 0.0309]).

#### 2.2.2. Neural tracking of Other.

**Fig 7:**
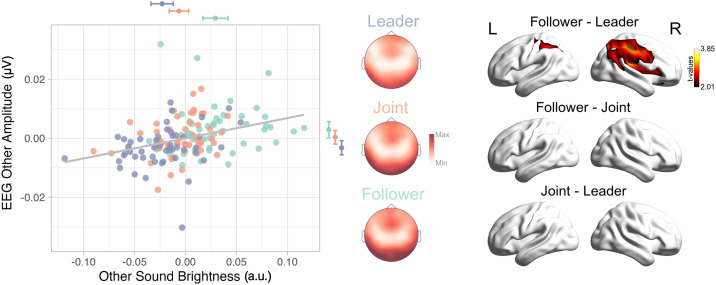
EEG Other responses as a function of sound brightness and leadership. The left panel shows the correlation between EEG Other responses and Other sound brightness (with line of best fit). Individual participants are shown in blue, red, and green for the Leader, Joint, and Follower conditions, respectively, with the corresponding means and 95% CIs computed for within-subject designs [72]. The middle column displays the grand-averaged topographic maps for the different leadership conditions represented on the same colour scale and the right column shows *t*-values of significant clusters for the pairwise comparisons between the three leadership conditions in the source space, indicating auditory and strengthened right hemisphere modulations. The EEG data underlying this figure are reported in S1 Data (Sheet 3).

***Effect of leadership*.** A repeated-measures ANOVA on the amplitude of EEG Other responses revealed a significant main effect of Leadership, *F*(2, 98) = 6.11, *p* = 0.004, *η*_*g*_^*2*^ = 0.02. Pairwise comparisons (3 tests in total) with Bonferroni correction indicated significantly larger Other response amplitude when following compared to when leading, *t*(49) = 3.25, *p* = 0.006, *d* = 0.42 (Fig 7). In contrast to the behavioural results for sound brightness, the difference between Leader and Joint did not reach significance, *t*(49) = 2.30, *p* = 0.08, *d* = 0.28. This dissociation suggests that the neural tracking of other-produced sounds was not only facilitated at peripheral level by changes in sound brightness, but also at central level by role-dependent selective enhancement mechanisms.

To further examine this possibility, regression analyses were conducted to assess whether leadership-related differences in EEG Other responses persisted after statistically controlling for variations in other-produced sound brightness (see Fig 7). As expected, the amplitude of EEG Other responses was positively correlated with Other sound brightness. However, a regression model that additionally included the factor Leadership accounted for more variance in EEG Other response amplitude, *F*(3, 146) = 8.60, *p* < 0.0001, *R*^*2*^ = 0.13, than a model including only Other sound brightness, *F*(1, 148) = 19.97, *p* < 0.0001, *R*^*2*^ = 0.11. Although the improvement in explained variance did not reach significance, *F*(2, 146) = 2.69, *p* = 0.07, the pattern of results is consistent with the ANOVA findings. Together, these results suggest that enhanced neural tracking of other-produced sounds when following is not driven solely by increased sound brightness, but may also be supported by centrally mediated, role-dependent gain mechanisms operating at higher levels of the auditory pathway.

Source space analyses (Fig 7, right column) further confirmed the effect of Leadership on the neural tracking of Other, with this effect being more pronounced in the right hemisphere. However, the overall response magnitude appears weaker than that observed for Self, as reflected by the fact that only the difference between Leader and Follower reached statistical significance.

The ANOVA on Other responses did not reveal any significant main effect of Sound Frequency, *F*(1, 49) = 0.10, *p* = 0.756, *η*_*g*_^*2*^ < 0.001, with comparable values across matched (M = 0.0203, 95% CI [0.0176, 0.0229]) and unmatched (M = 0.0207, 95% CI [0.0181, 0.0233]) conditions. Similarly, no significant interaction between Leadership and Sound Frequency was observed, *F*(2, 98) = 0.19, *p* = 0.821, *η*_*g*_^*2*^ < 0.001 (Follower–Matched: M = 0.0232, 95% CI [0.0194, 0.0271]; Follower–Unmatched: M = 0.0236, 95% CI [0.0202, 0.0269]; Joint–Matched: M = 0.0211, 95% CI [0.0171, 0.0239]; Joint–Unmatched: M = 0.0205, 95% CI [0.0171, 0.0239]; Leader–Matched: M = 0.0165, 95% CI [0.0140, 0.0189]; Leader–Unmatched: M = 0.0180, 95% CI [0.0143, 0.0218]).

#### 2.2.3. Self-Other neural integration/segregation.

**Fig 8:**
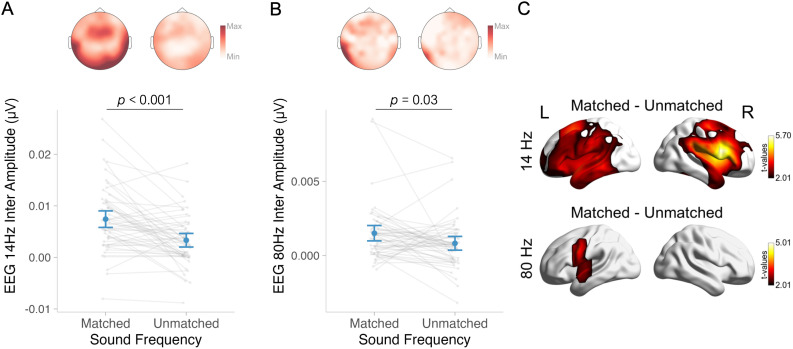
EEG intermodulation responses for matched and unmatched sound frequency. EEG Intermodulation responses at 14 and 80 Hz across sound frequency conditions with their corresponding grand-averaged topographic maps (**A** and **B**, presented on the same colour scale) and differences in the source space based on *t*-values of significant clusters **(C)**. Error bars represent 1 × 95% CI of the mean computed for within-subject designs [72]. Light grey lines represent individual participants. The EEG data underlying this figure are reported in S1 Data (Sheet 3).

A repeated-measures ANOVA on the EEG amplitude at 14 Hz and at 80 Hz, with the factors Leadership (Leader, Follower, and Joint) and Sound Frequency (Matched and Unmatched), indicated a significant main effect of Sound Frequency at both frequencies (14 Hz: *F*(1, 49) = 21.40, *p* < 0.001, *η*_*g*_^*2*^ = 0.06; 80 Hz: *F*(1, 49) = 4.97, *p* = 0.03, *η*_*g*_^*2*^ = 0.02). The amplitude at these frequencies, taken here as a proxy for self-other integration, was lower in the Unmatched compared to the Matched sound frequency condition, as depicted in Fig 8. Source space analyses confirmed this effect, showing a stronger right-hemispheric involvement for the 14 Hz response, whereas no clear hemispheric asymmetry was observed at 80 Hz. While caution is warranted given that 80 Hz responses were generally less reliable, these results suggest that the effect of distinct sound frequencies was generally more bilaterally distributed for the intermodulation frequencies than for self and other frequencies. Together, these results show that unmatched sound frequencies across paired participants, which improved interpersonal synchronisation relative to matched frequencies, also facilitated the neural segregation of self and other.

The ANOVA revealed no significant main effect of Leadership for either 14 Hz, *F*(2, 98) = 0.21, *p* = 0.778, *η*_*g*_^*2*^ = 0.001 (Follower: M = 0.0052, 95% CI [0.0034, 0.0067]; Joint: M = 0.0058, 95% CI [0.0041, 0.0074]; Leader: M = 0.0052, 95% CI [0.0036, 0.0067]) or 80 Hz, *F*(2, 98) = 0.89, *p* = 0.412, *η*_*g*_^*2*^ = 0.004 (Follower: M = 0.0009, 95% CI [0.0005, 0.0014]; Joint: M = 0.0013, 95% CI [0.0008, 0.0019]; Leader: M = 0.0012, 95% CI [0.0007, 0.0018]), and no significant interaction between Leadership and Sound Frequency for 14 Hz, *F*(2, 98) = 1.32, *p* = 0.271, *η*_*g*_^*2*^ = 0.006 (Follower–Matched: M = 0.0071, 95% CI [0.0047, 0.0096]; Follower–Unmatched: M = 0.0033, 95% CI [0.0014, 0.0052]; Joint–Matched: M = 0.0086, 95% CI [0.0065, 0.0107]; Joint–Unmatched: M = 0.0029, 95% CI [0.0012, 0.0045]; Leader–Matched: M = 0.0065, 95% CI [0.0045, 0.0085]; Leader–Unmatched: M = 0.0038, 95% CI [0.0020, 0.0056]). A significant interaction was observed for 80 Hz, *F*(2, 98) = 3.37, *p* = 0.040, *η*_*g*_^*2*^ = 0.018 (Follower–Matched: M = 0.0013, 95% CI [0.0006, 0.0019]; Follower–Unmatched: M = 0.0006, 95% CI [0.00003, 0.0012]; Joint–Matched: M = 0.0021, 95% CI [0.0013, 0.0029]; Joint–Unmatched: M = 0.0006, 95% CI [0.00004, 0.00119]; Leader–Matched: M = 0.0011, 95% CI [0.0004, 0.0018]; Leader–Unmatched: M = 0.0013, 95% CI [0.0006, 0.0020]). However, pairwise comparisons across all individual conditions (15 tests in total) with Bonferroni correction did not reveal reliable differences (all *p* values ≥ 0.08).

## 3. Discussion

This study provides evidence for a central role of self-other differentiation during auditory-based interpersonal coordination. It shows how the self-other distinction may be achieved along the human auditory pathway through mechanisms supporting the selective processing of self- and other-related information. Assigned leadership roles and distinguishable sounds, together with selective brain-based neural enhancement, appear to support the segregation of concomitant self and other acoustic signals at peripheral and central levels, thereby contributing to precise action coordination between individuals.

### 3.1. Designated leadership roles

Designated leader and follower roles facilitated interpersonal synchronisation through sound. Leadership roles are used in everyday auditory-guided cooperative activities, often emerging spontaneously if not explicitly designated [4,76]. This is the case in turn-taking behaviours, which are fundamental for successful conversations [77], for instance, where alternating leadership roles in speaker-listener relationships prevents the masking of self- and other-produced speech, and thus, facilitates understanding [78,79]. Turn-taking behaviours feature analogously in musical ensemble performance, where the melodic leader role can switch between parts [4,80,81].

Here we show that leader-follower roles are associated with facilitated neural processing of self- and other-produced sounds and help to achieve better temporally synchronised cooperative actions. Our results suggest that these roles contribute to mitigating the issue of masking inherent to the auditory modality by enhancing the neural processing of behaviourally relevant information in the acoustic environment, specifically the sounds produced by the leader, who is responsible for driving the interaction. We observed selectively enhanced neural tracking of the sounds generated by the leader in both the participant leading and the participant following, as shown in Figs 5 and 7. Expectations regarding who will initiate the changes may have an important function by allowing attention to be dynamically oriented towards relevant acoustic information and boost its neural processing, especially in the context of improvised actions where dynamic changes are not pre-planned and are hence largely unpredictable. This selective enhancement may counteract the process of self-attenuation that is often the focus of research on the self-other distinction [26,82]. These results thus provide new insights into the neural mechanisms that are exploited in leader-follower conventions adopted by humans to support effective coordination in everyday auditory-based cooperative activities [83].

### 3.2. Distinguishable sounds

Our results provide evidence that distinguishable acoustic features play a role in achieving precisely synchronised cooperative actions through sound. Specifically, leaders produced sounds that were more distinguishable by increasing their overall brightness facilitating their neural processing. Such behavioural modification can be viewed as a form of ‘coordination smoother’, previously documented in movement timing and amplitude [84,85], and here reflected in the salience of spectral cues. Leaders enhanced the brightness of their sounds in the current settings via increased overall finger pressure resulting in sounds that had more energy in higher frequencies (due to greater squareness of the waveforms). Increased brightness also results in sounds that are perceived as more salient [86], which might have further contributed to make leaders’ sound more distinguishable and improve synchronisation.

Independently of leadership roles, the results also indicate that paired participants achieve better synchronisation when the sounds they produce have different fundamental frequencies, and thus again, are more distinguishable. This finding is consistent with behavioural research focussing on the coordination of discrete sequential actions, such as experiments manipulating footstep sounds during walking and tone pitches during music production [5,14], extending the phenomenon to the domain of continuous behaviour. Furthermore, we shed light on its brain mechanisms by showing that the improvement in synchronisation is supported by enhanced neural tracking of self-produced sounds as well as decreased integration of self- and other-produced sounds. Our results suggest that unmatched fundamental sound frequencies facilitate interpersonal synchronisation by increasing an individual’s capacity to monitor changes in the sound they produce and separate them from those produced by their partner. Together, these results further our understanding of the mechanisms and sound features that humans exploit to successfully lead and interact with each other through sound. Changing sound brightness, loudness, and frequencies in order to be more distinguishable, and potentially enhance leadership and even attractiveness, has been previously documented in humans as well as animals [15,22,87]. The present findings go beyond previous research by demonstrating that these adjustments to acoustic features are an effective behavioural strategy for improving cooperative actions by selectively facilitating the underlying neural processing of self- and other-produced sounds.

### 3.3. Self-other processing hierarchy

We argue that designated leadership roles and distinguishable sounds, together with selective cortical neural enhancement, are complementary mechanisms that enable dynamic balancing of self-other integration/segregation at peripheral and central level along the human auditory pathway. Results from cochlear model simulations [88–94] (presented in Supporting information) to further examine effects of sound frequencies highlight the importance of peripheral constraints on the process of self-other distinction. They suggest that decreased self-other integration, as recorded with EEG in the form of decreased intermodulation frequencies elicited by unmatched sound frequencies, is driven by low-level peripheral processes. Sounds with matched fundamental frequencies are integrated at the level of the cochlea already, as they get processed in the same cochlear channel, in contrast to sounds with unmatched fundamental frequencies that are predominantly processed in separate cochlear channels. Remarkably, the results of cochlear modelling also highlight the importance of facilitatory mechanisms at the central level. They suggest a decrease of Self and Other amplitude at the cochlear level with different sound frequencies while our EEG results revealed no change in amplitude for Other and even a significant increase for Self.

Our EEG results further suggest that the right hemisphere compared to the left, and Self compared to Other, benefit most from distinct sounds and leadership roles. While methods with higher spatial resolution, such as MEG with individual brain templates [95,96], will be needed to confirm this lateralisation in future research, stronger effects in the right hemisphere may be due to its superiority for processing spectral information, providing an advantage for self-other differentiation [39,40]. Interestingly, converging evidence from previous studies also points to a right hemisphere dominance for self-related processing, both in auditory self-representations and in the sense of self more generally [29,41–43]. This is consistent with the stronger effects we observed for Self compared to Other, particularly in the right hemisphere, suggesting a critical role of the self-other distinction in maintaining control over one’s own actions and preventing agency confusion [97,98]. Together, these findings support a hierarchical account of self-other processing, with selective brain mechanisms and behavioural strategies (e.g., increasing overall brightness) at a central level working dynamically alongside peripheral constraints at the cochlear level to support optimal self-other integration/segregation, and thus, action coordination through sound.

### 3.4. Dual-EEG audio tagging in future research

The present findings highlight the strengths of combining dual-EEG and frequency tagging to investigate the fundamental behavioural and brain mechanisms that enable humans to interact through sound. This approach allowed us to record, with high signal-to-noise ratio, the neural processing of self- and other-produced sounds while preserving sufficient behavioural freedom to characterise the mechanisms participants used to achieve successful synchronisation. Crucially, this method not only enables the tracking of self- and other-specific processes, but also provides a direct index of their degree of integration, capturing a range of interconnected yet partially independent mechanisms along the auditory pathway [39,99–101]. For instance, leadership-based selective neural enhancement of self- and other-related information did not appear to extend to their integration. Additionally, the reduction in intermodulation-related responses associated with distinct fundamental sound frequencies was accompanied, not by a decrease but by an actual increase in self-related responses, highlighting the dissociation between self-, other-, and integrative processing.

Importantly, this method departs from conventional hyperscanning approaches, which typically focus on correlational or synchrony‑based measures computed directly between the neural activities of interacting individuals [102–105]. Such measures, primarily capturing temporal alignment across brains, make it difficult to dissociate cognitive and perceptual processes related to self-other distinctions from the behavioural synchrony and motor coupling that often accompany coordinated action [53–55]. This limitation becomes especially pronounced in highly dynamic synchronisation contexts, such as those investigated in the present study, where motor alignment and shared sensory inputs are intrinsically confounded with higher‑level social and cognitive processes.

By contrast, dual‑EEG audio frequency tagging enables the selective isolation of self‑ and other‑related sensory and cognitive processes, independently of motor activity, thereby providing a more direct way to investigate the mechanisms underlying joint action. To our knowledge, this study provides the first demonstration that this new methodological approach can be leveraged to disentangle self-other processing during interpersonal coordination in the auditory domain. In doing so, it extends our previous work using dual-EEG frequency tagging in the visual modality [52], and suggests that auditory interpersonal coordination relies on partially distinct processes.

Notably, the central role of the self-other distinction observed here contrasts with findings observed by Varlet and colleagues (2020) [52] using a similar synchronisation paradigm in the visual domain, where self-other integration, facilitated by movement similarity and undesignated leadership roles, was prioritised to improve interpersonal coordination. While future research directly comparing modalities will be needed to confirm and generalise this pattern, the present results suggest that self-other integration and segregation may be uniquely constrained and balanced across sensory modalities [106–108]. Such modality-specific constraints may influence how information exchange and action coordination are established and maintained between individuals [14].

Beyond these specific findings, these methodological and theoretical advances provide a promising framework for gaining a deeper understanding of self-other processes underlying everyday social coordination that are inherently multisensory [105,109–114]. This framework also offers new opportunities to investigate the interactional and communicative impairments associated with mental disorders and related conditions. It has direct relevance to understand interpersonal coordination disorders reported in patients with autism, schizophrenia, and social phobia [115–120]. More generally, a substantial body of evidence indicates that atypical auditory self-other processing is closely associated with auditory‑verbal hallucinations in schizophrenia and subclinical populations, as well as with broader disruptions of the sense of self across a range of clinical conditions [41,121–123]. The present framework therefore opens new avenues for examining the mechanisms affected in these populations, more specifically how the brain might atypically process and integrate/segregate self- and other-related information within and across sensory modalities. As such, this approach is well suited to shed fresh light on fundamental aspects of human (mis)communication, across verbal and nonverbal domains, directly relevant for the transfer of meaning and affective information between individuals at societal and cultural scales.

### 3.5. Conclusion

The present results suggest that the balance between self-other integration and segregation is supported along the auditory pathway by dynamic and complementary mechanisms, with distinct sounds and leadership conditions playing a key role. Such mechanisms may help humans compensate for self-other masking that can occur with acoustic signals, and may therefore differ from those previously observed in visually-guided interactions, where coordination appears to rely more strongly on self-other integration through action and movement similarity. Together, these findings advance our understanding of the behavioural and brain processes exploited by humans to support information exchange and communication in everyday auditory-based interactions and leader-follower conventions.

## 4. Materials and methods

### 4.1. Ethics statement

The study was approved by the ethics committee of Western Sydney University (approval number H13092) and has been conducted according to the principles expressed in the Declaration of Helsinki. Participants provided both written and verbal consent before participating.

### 4.2. Participants

Twenty-five pairs of participants (50 individuals in total) volunteered to take part in the study (32 females and 18 males aged from 19 to 46 years; *M* = 25.10, *SD* = 5.06). Our sample size was chosen based on an a priori power analysis to detect medium to large effect sizes (d = 0.5–0.8) with at least 80% power, consistent with effect sizes previously reported in EEG frequency tagging and interpersonal coordination literature [52,74,75]. Dyads were randomly assigned and comprised 11 female-female, 4 male-male, and 10 female-male pairs, including both previously acquainted and unacquainted participants. Musical experience varied across the sample, with 27 participants reporting previous musical training, distributed across 8 dyads with two musically trained participants, and 11 dyads with one trained participant. All participants were right-handed and had normal hearing.

### 4.3. Auditory stimuli

The auditory stimuli were generated in real-time using a custom-made C++ application with Xcode and the Openframeworks library on a MacBook Pro. The stimuli consisted of two continuous tones, one for each participant, delivered to the two participants binaurally at a sampling rate of 44.1 kHz via insert earphones (ER-2; Etymotic Research).

The sound brightness of Person 1 and Person 2 was modulated in real-time for each pair based on the force that participants exerted with their right index finger on a force sensor (FlexiForce A401, Tekscan, Inc, MA, US). Force signals were recorded at 60 Hz using an Arduino Duemilanove board (Arduino, Ivrea, Italy) and processed by the C++ application to control online adjustments of sound brightness with minimal latency (17 ms maximum latency). The sound brightness was manipulated by linearly mapping the shape of the audio signal to the force exerted, with zero exerted force corresponding to a perfect sinusoidal waveform and maximum exerted force (>111 N) corresponding to a perfect square waveform. Square waveforms result in adding harmonics, whose amplitude increases as the squareness increases, and thus, in brighter sounds. The linear matching was selected as the most straightforward and intuitive implementation consistent with everyday sensorimotor mappings, and was confirmed during pilot testing to be effective and easily understood by participants.

The fundamental sound frequency of Person 1 and Person 2, which remained constant throughout each trial, was either 200 Hz or 500 Hz, depending on the experimental condition. These frequencies (200 and 500 Hz) were selected for both methodological and ecological reasons. Low carrier frequencies are known to elicit robust, high signal‑to‑noise ratio EEG responses, while remaining within an ecologically valid range for vocal sounds [57–60]. Using relatively low carrier frequencies was also important for effective perceptual manipulation of sound brightness, as changes in waveform shape from sinusoidal to square waves yield salient timbral differences at low fundamental frequencies, whereas these differences become less perceptually distinct at higher frequencies, likely due to a coarser auditory encoding of the spectral envelope and increased interaction between pitch and brightness cues [124,125]. The spacing between the two carrier frequencies therefore reflected a compromise between perceptual discriminability and neurophysiological sensitivity, with frequencies sufficiently separated to be easily distinguishable while remaining low enough to preserve strong EEG response amplitudes and reliably perceived sound brightness modulations.

Finally, the envelopes of the sounds produced by Person 1 and Person 2 were sinusoidally modulated (modulation index 1 [126]) at 33 Hz and 47 Hz, respectively, prior to binaural presentation. This frequency tagging approach enabled the separate recording, with high-signal-to-noise ratio, of neural responses specific to self- and other-generated sounds, and their integration, despite continuous changes in participants’ exerted force and corresponding changes in the acoustic signals [52,64,127].

The modulation frequencies were selected because they reliably elicit auditory steady-state EEG responses while being sufficiently separated in the frequency-domain to allow unambiguous dissociation of self-related, other-related, and intermodulation components, and to minimise overlap with low-frequency neural oscillations and movement-related artefacts [67]. In addition, choosing modulation frequencies below 50 Hz ensured that the resulting higher-order intermodulation frequency (80 Hz) remained well below 100 Hz, thereby avoiding higher frequency ranges where EEG signal-to-noise ratios are reduced [128]. Recording reliable intermodulation frequencies was critical, as these components—emerging from the concurrent processing of both amplitude-modulated signals—provided a direct index of self-other integration [64,129,130]. Importantly, the 33 and 47 Hz tagging signals were phase-locked across trials, enabling averaging prior to frequency-domain analyses to further enhance the signal-to-noise ratio of self-related, other-related, and intermodulation responses while attenuating nonphase-locked EEG activity [128].

### 4.4. Experimental procedure and design

Upon arrival, the two participants were provided with an information sheet that described the auditory-based improvisation synchronisation task, as well as the EEG and force sensor equipment used. Written informed consent was then invited and obtained from all participants.

The two participants were then fitted with the EEG caps and electrodes and seated on two chairs positioned back-to-back preventing visual contact. They were equipped with the insert earphones and instructed to place their right index finger on the force sensor with their forearm resting comfortably on a custom-made support attached to the chair. Participants were shown that the timbral brightness of their continuous sounds could be changed by varying the downward finger force exerted on the sensor. Each participant completed an individual practice phase to familiarise themselves with the force-to-sound mapping, which was readily adopted by all participants within a few seconds.

Participants were then given instructions for the auditory-based improvisation synchronisation task. They were instructed to produce novel and dynamic modulations in sound brightness and to synchronise these modulations as closely as possible with those of their partner. Novelty could be achieved through continuous variations in both the magnitude and temporal structure of brightness changes, allowing participants to generate a wide range of distinct patterns over time. This was implemented in a self-paced manner, with no external metronome or timing cue, such that participants freely determined how to produce these dynamic changes while maintaining synchrony with their partner. This was followed by a brief practice phase in which both sounds were presented binaurally via the earphones, allowing participants to experience the combined auditory feedback. Participants then completed two practice trials in randomly selected experimental conditions before beginning the main task.

The participants were instructed to perform the task under three different Leadership conditions, as in the Varlet and colleagues (2020) [52] visual coordination study—the Leader-Follower condition, in which Person 1 was instructed to lead the improvisation; the Follower-Leader condition, in which Person 2 was instructed to lead the improvisation; and the Joint condition, in which no leader and follower roles were designated, and participants were instructed to perform together. In the conditions with a leader, the designated leader was instructed to initiate and shape the temporal dynamics of the sound modulations, while the other participant adapted their behaviour to maintain synchronisation. The three Leadership conditions were mixed with four different Sound Frequency conditions—two matched and two unmatched sound conditions, in which Person 1 and Person 2 had the same fundamental frequencies (200–200 Hz or 500–500 Hz) or different fundamental frequencies (200–500 Hz or 500–200 Hz).

Each dyad completed 24 trials of 180 s (i.e., 2 trials per condition across the 12 Leadership [3] × Sound Frequency [4] combinations), presented in a randomised order, yielding a total of 72 min of recorded interaction. Including EEG preparation and breaks, the total duration of the experimental session was ~100 min.

### 4.5. Behavioural analyses

Sound brightness data bounded by 0 (i.e., minimum force and brightness [perfect sinusoidal wave]) and 1 (i.e., maximum force and brightness [perfect square wave]) were used to compute participants’ mean sound brightness and the synchronisation between the dynamic modulations produced by the two participants using coherence analyses in each condition. Squared cross-spectral coherence was calculated from 0 to 5 Hz using Fast Fourier Transformation (FFT) windows of 10 s with 50% overlap, giving a value between 0 and 1 for each frequency bin, with 0 indicating no synchronisation and 1 indicating perfect synchronisation [68,69].

### 4.6. EEG recording and analyses

EEG was recorded at a sampling rate of 2,048 Hz using a Biosemi Active-Two system (Biosemi, Amsterdam, the Netherlands) with 64 Ag-AgCl electrodes placed over the scalp of each of the two participants according to the international 10/20 system. All electrodes were referenced to the Common Mode Sense (CMS) and their magnitude was kept below 50 mV. The Biosemi ActiveTwo system enabled simultaneous recording from both participants using a shared acquisition setup and integrated digital triggers marking the onset of each trial, ensuring precise temporal alignment of the EEG data across participants and within each experimental block.

Data were first high-pass filtered using a 4th order Butterworth filter with a cut-off frequency of 0.1 Hz to remove slow drifts, and notch filtered to remove 50 Hz (and its harmonics) electrical power contamination. The data were subsequently downsampled to 1,000 Hz and segmented into 180 s trials corresponding to the 180 s sequences of joint improvisation. Channels containing excessive artefact or noise were then interpolated with the neighbouring channels (i.e., an average of 0.65 [*SD* = 0.88] interpolated electrodes per participant and never more than 3 electrodes). An independent components analysis (FastICA), as implemented in Fieldtrip [95], was used to remove components corresponding to eye blink and lateralised eye movements based on visual inspection of their topography and time-course. EEG data were then re-referenced to the average of all scalp electrodes.

At the next stage of data processing, the two trials of each condition were averaged together to improve signal-to-noise-ratio as tagging periodic signals were time-locked between trials [66,73]. We then used a FFT on the average trial of each condition to compute for each EEG channel and participant the amplitude spectra up to 100 Hz with a frequency resolution of 0.0055 Hz (i.e., 1/180).

To examine the occurrence of significant EEG responses at Self (33 and 47 Hz for Person 1 and Person 2, respectively), Other (47 and 33 Hz for Person 1 and Person 2, respectively), and 14 and 80 Hz Intermodulation frequencies, we computed z-scores at each frequency bin after pooling together the spectra of all EEG channels. This averaging reduces dimensionality and increases the signal‑to‑noise ratio by reinforcing responses that are consistent across channels [52,75]. For each frequency of interest, z-scores were calculated as the difference between the amplitude at that frequency bin and the mean of the 20 neighbouring frequency bins (excluding the two immediately adjacent frequency bins), divided by the standard deviation of those 20 neighbouring bins. EEG responses were considered statistically significant when z-score values were greater than 1.96 (*p* < 0.05), in line with previous studies that used frequency tagging techniques [131,132], indicating signal amplitude reliably larger than the noise background.

Z-scores were computed both after averaging all participants (Person 1 and Person 2 separately) to assess overall significance at each frequency, and at the individual participant level to evaluate response reliability across the sample [52]. Whereas Self, Other, and Intermodulation frequencies elicited robust and consistent EEG responses across participants, higher harmonics were characterised by markedly lower amplitudes and limited cross‑participant consistency (≤50%) and were thus not considered in subsequent analyses.

To compare the amplitude of EEG responses at Self, Other, and Intermodulation frequencies across the different conditions, while controlling for the effect of background noise (including muscular artefacts), we subtracted at each frequency bin of the amplitude spectra the average amplitude of the 20 neighbouring frequency bins excluding the two immediately adjacent frequency bins [52,75]. This baseline subtraction procedure allowed background noise and muscular artefacts to be further controlled, since such irregularities affect amplitude spectra over a large range of frequency bins around those of interest. The obtained baseline-subtracted amplitude spectra were further averaged across all EEG channels for each participant and condition, and kept for further statistical analyses.

### 4.7. Source space analyses

Cortical responses phase-locked to the tagging signals and their intermodulation frequencies were source reconstructed for each participant using DICS [133]. We reconstructed the activity of pairs of dipoles, where left/right symmetric dipole pairs were used as a source model, as steady-state auditory stimulation often induces simultaneous (i.e., zero-lag correlated) activation in bilateral auditory areas [62,96]. We used a template standard head model (Montreal Neurological Institute; MNI) with a 1 cm grid [95]. Common special filters with 14, 33, 47, and 80 Hz dummy signals were computed for each voxel with 5% regularisation based on the cross-spectral density matrix obtained from FFT on data of all conditions segmented into 1 s epochs (50% overlap) with a Hanning window. Figures of source distributions (Figs 5 – 8) were made using BrainNet [134].

### 4.8. Statistical analyses

Conditions with identical sound-frequency pairings (200–200 and 500–500 Hz) and with different sound-frequency pairings (200–500 and 200–500 Hz) were averaged to form two Sound Frequency conditions—Matched and Unmatched. Two-way repeated-measures ANOVAs with the factors Leadership (Leader, Follower, and Joint) and Sound Frequency (Matched and Unmatched) were then conducted on participants’ mean sound brightness, and EEG amplitudes at the Self (33 and 47 Hz for Person 1 and Person 2, respectively), Other (47 and 33 Hz for Person 1 and Person 2, respectively), and 14 and 80 Hz Intermodulation frequencies. Individual-level conditions in these analyses were thus based on a total of 12 min of continuous interaction, providing reliable behavioural and neural estimates.

In contrast, interpersonal behavioural coherence was analysed at the dyadic level. Leader-Follower and Follower-Leader conditions were therefore averaged to form two Leadership conditions—Designated and Undesignated—and analysed using a two-way repeated-measures ANOVA with the factors Leadership (Designated and Undesignated) and Sound Frequency (Matched and Unmatched). This averaging reflects the inherently dyadic nature of the coherence measure, with each resulting condition represented by a total of at least 12 min of continuous interaction, enabling reliable estimation of interpersonal synchronisation.

All statistical analyses were conducted in R version 3.4.3 [135] and graphics were made with the package ggplot2 [136]. Repeated-measures ANOVAs were performed using the package “afex” version 0.19-1 [137] with Greenhouse-Geisser correction applied when the assumption of sphericity was violated. Pairwise contrasts were used to explore the significant effects further, with Bonferroni adjustment for multiple comparisons. Linear regression analyses were also conducted using the standard stats package of R to further explore the links between different dependent variables. Statistical differences between conditions in source space were examined using cluster-based permutation testing with two-tailed paired *t*-tests and 1,000 permutations, as implemented in Fieldtrip [95]. Confidence intervals presented in the text and figures (as error bars) are computed as within‑subject 95% confidence following the method described by Morey (2008) [72], which appropriately accounts for the dependency structure inherent in repeated‑measures designs. This approach ensures that the confidence intervals reflect within‑participant variability rather than between‑participant differences, providing a more accurate representation of condition effects.

## Supporting information

S1 File Auditory modelling: effects of brightness and carrier frequency-matching on subcortical auditory processing. Biologically plausible auditory simulations were used to model subcortical processing of the experimental sound mixtures in Matched and Unmatched conditions. Responses to synthetic amplitude-modulated tone pairs were simulated at the auditory nerve and inferior colliculus, and frequency-domain analyses were applied to quantify modulation and intermodulation responses for comparison with EEG findings.
(PDF)

S1 Data Data underlying the figures and statistical analyses reported in the manuscript. Sheet 1 contains mean coherence values for each dyad. Sheet 2 contains mean sound brightness values for each participant. Sheet 3 contains baseline-subtracted EEG amplitudes at the self, other, 14-Hz and 80-Hz frequencies for each participant. Data are reported as a function of leadership condition and sound frequency condition.
(XLSX)

## Abbreviations

CMS: Common Mode Sense
DFT: discrete Fourier transform
DICS: Dynamic Imaging of Coherent Sources
FFT: Fast Fourier Transformation.
