# Supplementary material for: Lateralised neural mechanisms facilitate auditory self-other distinction and coordination in human dyads
Source: PLoS Biol. 2026 Aug 10;24(8):e3003935. doi: 10.1371/journal.pbio.3003935 (PMC13475979; doi:10.1371/journal.pbio.3003935)
Supplement: Supplementary file 1 [file pbio.3003935.s001.pdf]

Auditory modelling: effects of brightness and carrier frequency matching on subcortical auditory processing

We used a biologically plausible model of subcortical auditory processing to examine the contributions of peripheral and higher-level mechanisms to EEG responses at the modulation and intermodulation frequencies in the Matched and Unmatched sound frequency conditions. We simulated responses to the sound mixture delivered to participants in the experiment (fundamental [200/500 Hz] and modulation frequencies [33/47 Hz]) at different stages of the auditory pathway. Because simulating responses to the actual sounds produced by participants would have been computationally expensive due to their duration (180 s per trial), we simulated responses to synthesised sounds with similar features.

These sound inputs consisted of 3-second sound snippets, which were generated by summing two tones 100% amplitude-modulated at 33 and 47 Hz. The tones had frequencies 200-200 Hz, 500-500 Hz, 200-500 Hz, and 500-200 Hz respectively, thus recreating the Matched and Unmatched conditions from the experiment. Furthermore, the simulation was repeated 100 times and random brightness was assigned to each constituent tone in the simulated pair (fixed across simulated conditions). The sound level was set to 75 dB SPL.

The synthetised sound mixtures were first passed through a model of the auditory nerve (AN) developed by Zilany et al. (88) as implemented in the UR_EAR toolbox (version 2020a). This model simulated responses from 128 cochlear channels with characteristic frequencies logarithmically spaced between 130 and 8000 Hz. The parameters used for cochlear tuning matched data available from human subjects (89). For each channel, 51 auditory nerve fibers were simulated with

biologically plausible distribution of high, mid, and low-spontaneous-rate fibers (90). The model provides faithful simulation of physiological processes associated with cochlear nonlinearities, inner hair cell transduction process, the synapse between the hair cell and the auditory nerve, and the associated firing rate adaptation.

The simulated auditory nerve firing rates were fed into the same-frequency inhibition and excitation model (SFIE) used to simulate enhanced onset synchrony and the decreased upper limit for phase-locking to stimulus envelope in the ventral cochlear nucleus (91). The default parameters in the UR_EAR toolbox were used, which were based on Carney et al. (92). A second SFIE model was then used to simulate band-pass modulation filtering and enhanced onset responses of neurons in the inferior colliculus (IC). The parameters were set to simulate a population of IC units tuned to the best modulation frequency at 40 Hz.

For both the AN and IC stage, the simulated instantaneous firing rates were summed across cochlear channels (93,94), and transformed into the frequency domain using FFT. The obtained spectra were normalized at each frequency bin by subtracting mean magnitude at neighbouring bins 2-3 on both sides. Response magnitudes at modulation and inter-modulation frequencies were extracted for each simulated response and averaged for the Matched and Unmatched conditions, in line with the EEG analysis.

Results of the simulations

The average magnitude at modulation frequencies (33 and 47 Hz) was compared across conditions. This is equivalent to the Self and Other responses, assuming no high-level effect of leadership. As shown in Fig A, the simulated response at modulation frequencies was consistently smaller in the Unmatched

condition at the level of the auditory nerve, which contrasts with our EEG results showing higher Self and similar Other responses in this condition. Hence, this simulation suggests that EEG results in the Matched and Unmatched conditions are not explained by early auditory nonlinearities but rather reflect higher-level selective enhancement of Self and Other responses in the Unmatched condition.

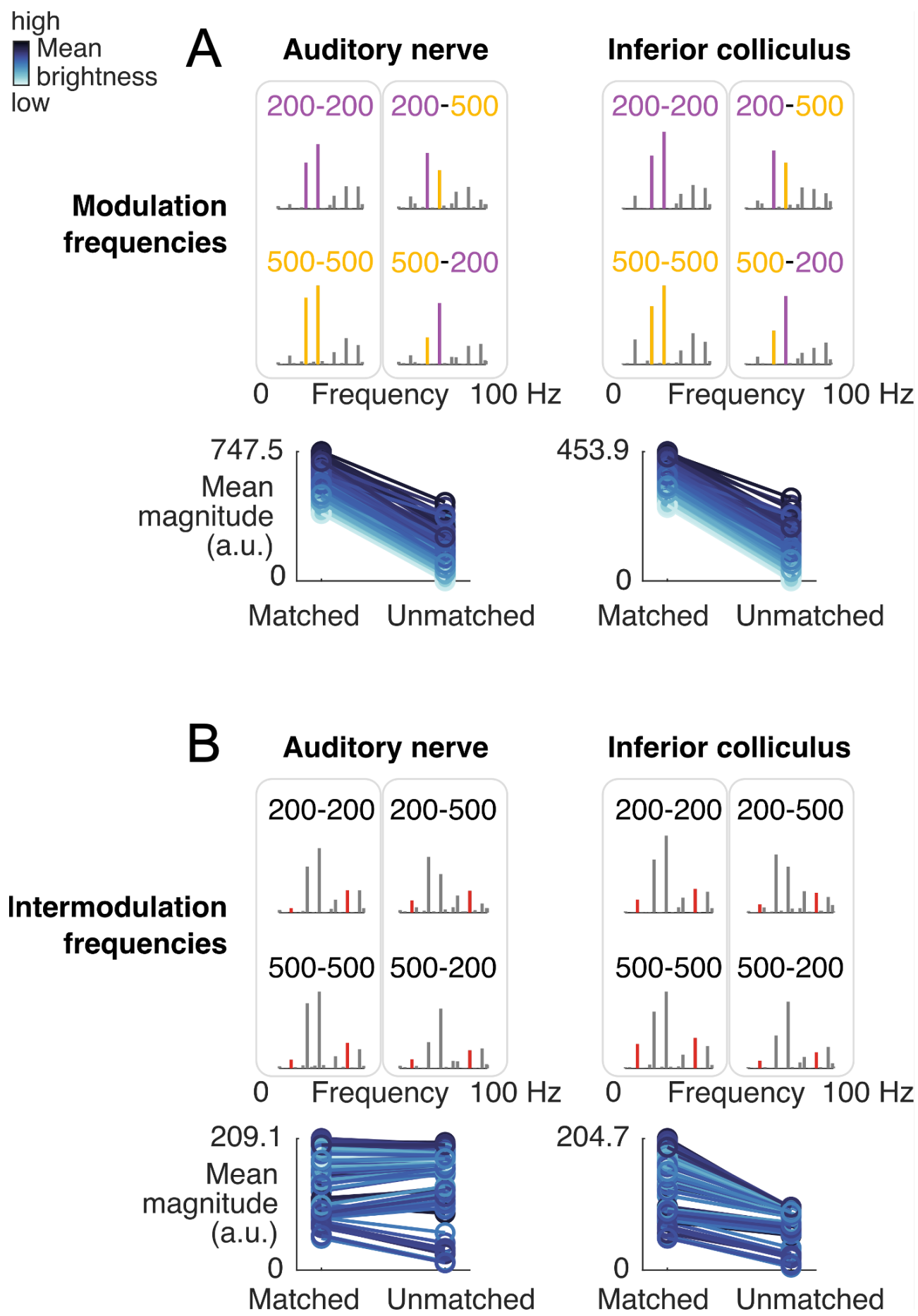

Fig A. Summary of the responses simulated with a biologically plausible model of the auditory periphery. Simulated responses from the auditory nerve are shown on the left and responses from the inferior colliculus on the right. (**A**) Responses at modulation frequencies. (Top) Spectra averaged across simulations with different amount of brightness assigned to the constituent sounds, separately for each condition. (Bottom) Extracted magnitudes for each condition. Each point represents a response to a single simulated 3-s audio snippet. The average brightness across the two simulated subjects in each audio snippet is shown as

colour gradient, and the responses to snippets with equivalent brightness levels are connected with lines across conditions. The response at modulation frequencies is enhanced in the Matched condition for the auditory nerve as well as the inferior colliculus. (**B**) Responses at intermodulation frequencies. The auditory nerve responds comparably across the Matched and Unmatched conditions. However, the response from the inferior colliculus is suppressed in the Unmatched condition.

Interestingly, the average simulated response at intermodulation frequencies was comparable between the Matched and Unmatched condition at the level of the auditory nerve. However, the inferior colliculus units consistently responded with larger magnitude at intermodulation frequencies for the Matched condition. This suggests that additional nonlinearities introduced in the later stages of the auditory pathway may partly explain the observed EEG results.

The physiological mechanisms driving the simulated responses at intermodulation frequencies are likely complex. One plausible mechanism giving rise to the effect could be (i) narrowband filtering at the cochlear level, (ii) followed by nonlinearity due to saturation. Both of these phenomena occur early in the auditory periphery and are closely reproduced in biologically plausible model (92). Specifically, in the Matched condition, the tones generated by each subject excite a single cochlear channel. Thus, the envelope containing both modulation frequencies will be extracted at the cochlear level. Subsequently, nonlinearities occur at the following subcortical stages, giving rise to prominent intermodulation frequencies. On the other hand, in the Unmatched condition, 200 and 500 Hz tone (and their harmonics) are partially resolved at the cochlear level. Therefore, the two constituent tones will not interact as strongly as in the Matched condition, and instead will be processed relatively separately within different frequency channels, which—after

passing through later nonlinearities—results in weaker intermodulation frequencies compared to the Matched condition.

Conclusion

Together, these simulations indicate that the differences in EEG responses at the modulation frequencies between the Matched and Unmatched conditions are likely driven by higher-level processes, which selectively amplify task-relevant information in the complex auditory scene. Indeed, cochlear simulations show larger amplitude at the modulation frequencies in the Matched condition compared to the Unmatched condition whereas EEG results show no difference between these two conditions for Other and even a significant increase for Self in the Unmatched condition, which supports the occurrence of higher-level selective neural enhancement. In addition, the simulations reveal that the intermodulation responses observed in the EEG responses may be partially explained by nonlinearities in the auditory periphery. These observations suggest that unmatched tones allow better differentiation between Self and Other, due to the separation of representations already in the auditory periphery (which is indexed by smaller intermodulation responses in the Unmatched condition). This early segregation appears to play an important role in the context of movement coordination when fed to higher-level cortical systems, in line with the current behavioural results.
