# Supplementary material for: Lateralised neural mechanisms facilitate auditory self-other distinction and coordination in human dyads
Source: PLoS Biol. 2026 Aug 10;24(8):e3003935. doi: 10.1371/journal.pbio.3003935 (PMC13475979; doi:10.1371/journal.pbio.3003935)
Supplement: Supplementary file 3 [file pbio.3003935.s006.docx]

Editor’s comments

As you will see below, the reviewers overall think that your study is well executed and provides important insights. However, they also raised a number of concerns about a lack of clarity, the statistical analyses and some potential confounders, including the small number of trials in each condition (Reviewer 2, point 2).

We thank the Editor for the positive evaluation of our study and for highlighting the reviewers’ constructive comments. We have carefully revised the manuscript to address all concerns raised, with particular attention to improving clarity, refining the methodological approach and analyses, and discussing potential confounding factors. All changes are highlighted in red in the manuscript and detailed below.

Specifically, we have clarified the experimental design and analytical approach prior to the presentation of the results to enhance readability. We also now systematically report all non‑significant effects alongside the corresponding means and confidence intervals for transparency. In addition, we have strengthened the theoretical background and framing in the Introduction and expanded the Discussion to provide a clearer interpretation of the results and their broader implications, as recommended.

Regarding the number of trials per condition, we have strengthened the justification of our design and clarified why the available data constitute a key strength of the study. First, we provide more details on the effective number of trials contributing to each condition. Specifically, the matched and unmatched conditions are each derived from the combination of two underlying frequency pairings (i.e., 200–200 and 500–500 for matched; 200–500 and 500–200 for unmatched), resulting in a minimum of four trials per condition contributing to the statistical analyses. Moreover, our approach relies on three‑minute continuous trials, yielding a total of 72 minutes of interaction per dyad across 24 trials. Statistical analyses are therefore based on at least 12 minutes of continuous recording per condition. Combined with a sample size of 25 dyads (50 participants), this provides a robust dataset supporting reliable behavioural and neural estimates, particularly in the context of the dual‑EEG frequency‑tagging approach, which is designed to maximise signal‑to‑noise ratio by leveraging extended stimulation periods.

We believe that these revisions have significantly strengthened the manuscript and hope that the Editor and reviewers will find that their concerns have been satisfactorily addressed.

------------------------------------
REVIEWS:

Reviewer #1: Thank you for the opportunity to read this very interesting and timely paper. I found it very well written and a valuable addition to research on social neuroscience of coordination. The findings have implications for broader work on communication and validate a novel paradigm that could be adapted to test these more ecologically-valid, complex interaction contexts. I have relatively minor comments below that I hope will add to the clarity of the manuscript and broaden its reach to multi-disciplinary audiences.

Introduction:
- It would be helpful for the reader to be more clearly guided through the Introduction, adding clear link from previous research to the focus of the current study. Can the research question be brought in earlier to help frame the background? The main study hypothesis is also presented in a long sentence of dense language- this would benefit from being split into two sentences with more concrete language: "We hypothesised that these mechanisms, together with those based on lower-level acoustic differences, constitute a powerful and flexible processing gradient enabling stream segregation at peripheral and central levels along the auditory pathway, key to maintaining the self-other distinction in everyday dynamic interactions characterised by temporally overlapping acoustic signals and uncertainty."

We thank the reviewer for this helpful suggestion. The Introduction has been substantially revised to provide a clearer and more structured progression from previous research to the focus of the current study. In particular, the research question is now introduced earlier in the Introduction to frame the background more explicitly and to guide the reader through the rationale of the study.

In addition, to improve clarity and readability, we have simplified the presentation of the study aims. Specifically, the original hypothesis statement—which was indeed expressed in an overly complex formulation—has been removed and replaced with a clearer articulation of the research question and study objectives. We believe that these changes enhance the accessibility, coherence, and overall structure of the Introduction.

- The definition for 'brightness'- the key manipulation for participants in this study is buried in the Figure 1 legend - please define this key term in the main text.

The term brightness is now explicitly defined in the main text. Methodological details have been added at the beginning of the Results section, including a clear explanation of the brightness manipulation and other essential methodological information (p. 6-7).

“*Twenty‑five pairs of participants performed the joint improvisation synchronisation task while EEG was recorded. Participants were seated back‑to‑back and could not see each other. Each participant controlled in real time a continuous tone via dynamic self-paced index‑finger pressure modulations recorded with a force sensor. The acoustic feature of the continuous tone modulated through finger pressure was* ***brightness****, defined as the perceived clarity or sharpness of a sound and primarily determined by the spectral centroid, that is, the relative proportion of sound energy in the higher harmonics or high‑frequency content (17,56). The brightness modulation was operationalised as a linear shift from a sinusoidal waveform at minimum force to a square waveform at maximum force, with increased squareness adding upper harmonics and producing a brighter sound.”*

Results: 
- The pattern is results is described before (and independent of) stats, which makes hard to match up.

Methodological details have been added at the beginning of the Results section to clarify the analytical procedures and measures. In addition, the description of observed patterns and the corresponding statistical results have now been brought together to ensure that readers can directly match each reported pattern with its supporting analysis.

- Some effects are not mentioned at all (eg. leadership x frequency on interpersonal synchronisation, or main effect and interaction on sound brightness)- I assume these were all non-significant, but at the very least they should be acknowledged as such in the text with maximal p-value.

All non‑significant effects for all independent variables are now explicitly reported in the Results section, including the main effects and interactions involving the factors Leadership and Sound Frequency. For each non‑significant result, we provide the corresponding test statistic, p‑value, and effect size.

- Plots only show significant effects, rather than means for the fully crossed designs- non-significant factors are excluded. Please show complete plots of fully crossed data for transparency.

Displaying full figures for all fully crossed designs would substantially reduce the clarity of the main results due to the number and visual complexity of the plots. To preserve readability, the figures in the manuscript therefore continue to focus on the significant effects.

However, to ensure transparency, we now report the corresponding means and confidence intervals for all non‑significant effects directly in the Results section. We believe that this approach provides sufficient detail while maintaining a clear and interpretable presentation of the main findings.

- Analysis of neural tracking- it's not clear why regressions are needed, they are presented as simply 'confirming' the ANOVA results?

The regression analyses were not intended to simply replicate the ANOVA results, but rather to provide a more direct test of the selective, leadership‑related top‑down neural enhancement of self‑ and other‑generated sounds by controlling for variance in neural responses attributable to brightness differences. We have added further methodological detail in the Results section to clarify the rationale for these analyses and to better articulate their contribution to interpreting the neural tracking of self‑ and other‑generated sounds (p. 14-15 and 17).

*“This dissociation suggests that the enhancement of neural responses to self-produced sounds cannot be fully explained by peripheral, stimulus-driven differences in acoustic input. Instead, it points to an additional contribution from centrally mediated mechanisms, consistent with selective enhancement of self-related auditory signals at the cortical level as a function of leadership roles.*

*To directly test whether leadership-related differences in Self responses could be explained by differences in sound brightness alone, we conducted multiple linear regression analyses. These analyses allowed us to statistically control for the contribution of peripheral acoustic variation while assessing the independent predictive value of leadership roles (Fig 5). This analysis showed that participants’ own sound brightness was a significant predictor of EEG Self response amplitude, t(146) = 4.97, p < 0.0001. Importantly, however, leadership roles also explained additional variance beyond sound brightness, with both Joint, t(146) = 3.36, p = 0.001, and Leader, t(146) = 2.73, p = 0.007, being significant predictors. Consistent with this interpretation, a model including the factor Leadership, F(3, 146) = 23.85, p < 0.0001, R^2^ = 0.32, explained significantly more variance, F(2, 146) = 6.30, p = 0.002, than a simpler model including only Self sound brightness, F(1, 148) = 55.02, p < 0.0001, R^2^ = 0.27.*

*Together, these regression results further indicate that leadership-related modulations of neural responses to self-produced sounds cannot be reduced to peripheral acoustic differences alone. Rather, they suggest that participants’ interactive roles contributed independently to the cortical weighting of self-generated auditory input, supporting the interpretation of a role-dependent, top‑down modulation of self-related auditory processing.”*

*“This dissociation suggests that the neural tracking of other-produced sounds was not only facilitated at peripheral level by changes in sound brightness, but also at central level by role-dependent selective enhancement mechanisms.*

*To further examine this possibility, regression analyses were conducted to assess whether leadership-related differences in EEG Other responses persisted after statistically controlling for variations in other-produced sound brightness (see Fig 7). As expected, the amplitude of EEG Other responses was positively correlated with Other sound brightness. However, a regression model that additionally included the factor Leadership accounted for more variance in EEG Other response amplitude, F(3, 146) = 8.60, p < 0.0001, R^2^ = 0.13, than a model including only Other sound brightness, F(1, 148) = 19.97, p < 0.0001, R^2^ = 0.11. Although the improvement in explained variance did not reach significance, F(2, 146) = 2.69, p = 0.07, the pattern of results is consistent with the ANOVA findings. Together, these results suggest that enhanced neural tracking of other-produced sounds when following is not driven solely by increased sound brightness, but may also be supported by centrally mediated, role-dependent gain mechanisms operating at higher levels of the auditory pathway.”*

- Currently behavioural and neural data are considered/analysed completed separately. Can additional analyses be run to test for links between these datasets, to support interpretation of the oscillations data?

Additional analyses were conducted to examine the relationship between neural tracking and behavioural synchronisation. Specifically, correlations were computed between EEG frequency‑tagged responses for each dyad (summed across participants) for self‑, other‑, and intermodulation components, and the degree of interpersonal synchronisation as measured by coherence. No significant correlations were observed.

These analyses are reported below as exploratory findings but were not included in the manuscript, as they remain inconclusive at this stage. This lack of association should be interpreted with caution, as the present sample size (selected to have sufficient power to detect hypothesised effects in our main analyses) may be insufficient to reliably detect brain–behaviour correlations at the level of inter-dyad differences. Indeed, such relationships are typically characterised by small effect sizes and are known to require substantially larger samples to achieve stable and reproducible estimates (e.g., Gell et al., 2024; Le Guern et al., 2026).

Method:
- Please justify your sample size and comment on power.

Our sample size has been justified in the Methods section (p. 28-29).

*“Our sample size was chosen based on an a priori power analysis to detect medium to large effect sizes (d = 0.5-0.8) with at least 80% power, consistent with effect sizes previously reported in EEG frequency tagging and interpersonal coordination literature (52,74,75).”*

- Did any of the dyads know each other before the experiment?

The study included a mix of dyads who knew each other beforehand and dyads who did not. This information has now been clarified in the Participants section (p. 29). Given the within‑subject nature of the analyses, familiarity between dyad members is held constant across conditions and is therefore unlikely to have systematically influenced the reported effects.

*“Dyads were randomly assigned and comprised 11 female-female, 4 male-male and 10 female-male pairs, including both previously acquainted and unacquainted participants.”*

Reviewer #2: This innovative study combined dual-EEG and frequency-tagging techniques in a more ecological dyadic improvisation synchronization task to investigate a relevant and relatively understudied question in auditory perception: how individuals discriminate between self- and other-generated sounds in joint auditory-motor synchronization. By integrating behavioral and neural measures, the study shows that auditory self-other distinction is modulated by both stimulus characteristics (spectral information) and participant's control over one's own actions (leader-follower roles). These findings have important implications for our understanding of the mechanisms underlying social coordination in auditory interactions. There are several laudatory aspects to highlight, including the innovative integration of dual-EEG and frequency tagging techniques, as well as the relatively large sample size for hyperscanning research (25 dyads). However, some methodological and conceptual issues temper confidence in some of the authors' claims. Addressing the points outlined below would substantially strengthen the manuscript.

A. Method: 
1. Participants: The manuscript does not report information on participants' musical training or rhythmic ability, which may represent a major confound for synchronization accuracy and sensitivity to changes in sound frequency (e.g., Scheurich et al., 2020). If these data were not collected, the authors should at least acknowledge this as a limitation and discuss the likely direction of its impact. Sample size justification should also be added to the manuscript.

Information on participants’ musical expertise was collected and is now reported in the Participants section (p. 29).

*“Musical experience varied across the sample, with 27 participants reporting previous musical training, distributed across 8 dyads with two musically trained participants, and 11 dyads with one trained participant.”*

Given the within‑subject design of the study, each participant contributed to all experimental conditions, such that stable individual differences—such as musical expertise—are held constant across conditions within participants. As a result, these factors are unlikely to systematically influence the observed effects, which are driven by within‑participant contrasts. While an interaction between musical expertise and the experimental factors cannot be fully excluded, our exploratory analyses did not reveal any clear or consistent effects of musical expertise on behavioural synchronisation performance or on neural responses

There was no significant difference in interpersonal coherence between pairs with at least one participant with musical training and those without. Similarly, no significant difference was observed when comparing pairs with two musically trained participants to those without.

Analyses of EEG responses also did not reveal any differences between participants with and without musical training for the overall Self, Other, 14 Hz, and 80 Hz responses.

As these effects were not central to the research questions of the present study, they are not included in the main analyses. However, information on participants’ musical expertise has been made available alongside the publicly shared behavioural and neural dataset, allowing future work to explore these effects in more depth.

Finally, we have now included a justification of the sample size in the manuscript based on an a priori power analysis (p. 28-29).

*“Our sample size was chosen based on an a priori power analysis to detect medium to large effect sizes (d = 0.5-0.8) with at least 80% power, consistent with effect sizes previously reported in EEG frequency tagging and interpersonal coordination literature (52,74,75).”*

2. Trial number: A critical limitation concerns the small number of trials in each condition. With only two trials per condition, the robustness of within-condition EEG estimates is likely reduced, despite the long trial duration (180 s each). This experimental choice requires justification. In addition, the authors should consider providing supplementary reliability analyses to increase confidence in the robustness of the data interpretation.

First, the effective number of trials contributing to each condition is greater than it may initially appear. Specifically, the matched and unmatched conditions are each derived from the combination of two underlying frequency pairings (i.e., 200–200 and 500–500 for matched; 200–500 and 500–200 for unmatched), resulting in a minimum of four trials per condition contributing to the statistical analyses.

Moreover, the use of three-minute long, continuous trials is a deliberate methodological choice. Across the experiment, we obtained a total of 72 minutes of interaction per dyad (i.e., at least 12 minutes of continuous recording per condition), yielding sizeable amounts of information‑rich data for both behavioural and neural measures. From a signal-processing perspective, what primarily constrains the signal-to-noise ratio in frequency-tagged EEG is the total amount of analyzable data rather than the nominal number of trials. In theory, averaging many short trials or fewer long trials can yield comparable signal quality when the total data duration is equivalent (Norcia et al., 2015).

In this context, we opted for longer, continuous trials because they are well suited to the dual-EEG frequency-tagging approach and the interactive behavioural paradigm employed here. Extended recordings support the resolution of stable frequency-domain responses while preserving the continuity of social interaction, which would be disrupted by frequent trial interruptions. We therefore refrained from fragmenting the data into a larger number of shorter trials, as this would have altered the ecological and interactive nature of the task.

To further address concerns regarding robustness, we have clarified in the manuscript how the amount of data contributing to each condition supports reliable estimation of both behavioural and neural effects, and we have revised the Methods and Results sections to make this rationale more explicit (p. 7, 33, and 36-37).

*“They then completed 24 three‑minute trials in which sound frequency and leadership roles were manipulated, resulting in a total of 72 minutes of recorded interaction across the experiment to ensure reliable behavioural and neural estimates.”*

*“Each dyad completed 24 trials of 180 s (i.e., 2 trials per condition across the 12 Leadership (3) × Sound Frequency (4) combinations), presented in a randomised order, yielding a total of 72 minutes of recorded interaction. Including EEG preparation and breaks, the total duration of the experimental session was approximately 100 minutes.”*

*“****4.8. Statistical analyses***

*Conditions with identical sound-frequency pairings (200 Hz – 200 Hz and 500 Hz – 500 Hz) and with different sound-frequency pairings (200 Hz – 500 Hz and 200 Hz – 500 Hz) were averaged to form two Sound Frequency conditions – Matched and Unmatched. Two-way repeated-measures ANOVAs with the factors Leadership (Leader, Follower, and Joint) and Sound Frequency (Matched and Unmatched) were then conducted on participants’ mean sound brightness, and EEG amplitudes at the Self (33 Hz and 47 Hz for Person 1 and Person 2, respectively), Other (47 Hz and 33 Hz for Person 1 and Person 2, respectively), and 14 Hz and 80 Hz Inter-modulation frequencies. Individual-level conditions in these analyses were thus based on a total of 12 minutes of continuous interaction, providing reliable behavioural and neural estimates.*

*In contrast, interpersonal behavioural coherence was analysed at the dyadic level. Leader-Follower and Follower-Leader conditions were therefore averaged to form two Leadership conditions – Designated and Undesignated – and analysed using a two-way repeated-measures ANOVA with the factors Leadership (Designated and Undesignated) and Sound Frequency (Matched and Unmatched). This averaging reflects the inherently dyadic nature of the coherence measure, with each resulting condition represented by a total of at least 12 minutes of continuous interaction, enabling reliable estimation of interpersonal synchronisation.”*

3. Another issue refers to the force-brightness mapping. The perceptual validity of the mapping between linear force and sound brightness is not demonstrated, leaving unclear whether "brightness" manipulations were perceived consistently across participants. More information is needed. For example, a pilot perceptual validation could demonstrate that participants reliably perceive increasing force as increased brightness.

The mapping between finger pressure and sound brightness was designed as a continuous and intuitive sensorimotor transformation, in which increases in force resulted in progressively brighter (i.e., more spectrally rich) sounds through controlled changes in waveform shape. While we did not conduct a formal perceptual validation study, several aspects of the design support the robustness of this mapping.

First, participants completed practice trials of sufficient duration (3 minutes) prior to the experiment, allowing them to familiarise themselves with the task and effectively calibrate the relationship between finger pressure and the resulting changes in sound. During pilot testing and the experimental sessions, all participants were able to rapidly learn and control this mapping, producing continuous and coordinated modulations in sound brightness.

Second, potential variability in the perception or use of the mapping across participants or dyads is unlikely to systematically affect the main results, given the within‑subject design of the study. Each participant contributes to all experimental conditions, such that any individual differences in sensitivity to the mapping are held constant across conditions and therefore do not confound the comparisons of interest.

Finally, we note that the mapping itself is not intended to reflect a natural acoustic relationship, but rather to provide a controlled and continuous parameterisation of sound brightness. Nevertheless, the association between perceptual brightness and higher spectral centroid is well established (Schubert & Wolfe, 2006; Wallmark, Nghiem, & Marks, 2021). As such, the ability of participants to reliably manipulate this dimension—rather than precise perceptual matching across individuals—is the critical requirement for the present study. We have clarified these points in the manuscript to better explain the rationale and implementation of the mapping (p. 7, 29-30, and 32).

*“The two auditory streams were presented together to each participant binaurally via insert earphones. Participants were given practice trials to familiarise themselves both with the mapping between finger‑pressure changes and corresponding changes in sound and with the synchronisation task.”*

*“The sound brightness was manipulated by linearly mapping the shape of the audio signal to the force exerted, with zero exerted force corresponding to a perfect sinusoidal waveform and maximum exerted force (> 111 N) corresponding to a perfect square waveform. Square waveforms result in adding harmonics, whose amplitude increases as the squareness increases, and thus, in brighter sounds. The linear matching was selected as the most straightforward and intuitive implementation consistent with everyday sensorimotor mappings, and was confirmed during pilot testing to be effective and easily understood by participants.”*

*“Each participant completed an individual practice phase to familiarise themselves with the force-to-sound mapping, which was readily adopted by all participants within a few seconds.”*

4. I am also concerned with potential motor contamination of EEG signals. The finger force manipulation used to control sound brightness may introduce motor-related artifacts that could influence the EEG measures (see, for example, Timm et al., 2014). The authors should justify how motor contamination was addressed or mitigated.

Potential motor contamination is precisely one of the key issues that motivated the use of the frequency‑tagging approach in the present study. By modulating the auditory signals at specific tagging frequencies (33 and 47 Hz), this method enables the selective isolation of neural responses that are strictly time‑locked to the tagged auditory envelopes, thereby dissociating them from broader neural activity associated with motor processes.

Critically, motor‑related activity is primarily expressed in lower‑frequency ranges and is not expected to generate narrow spectral peaks at the tagging frequencies. The frequency‑domain analyses confirmed clear and specific peaks at the tagging frequencies and their intermodulation components (see Fig 4), indicating that the measured responses predominantly reflect auditory processing of self‑ and other‑generated sounds rather than motor‑related fluctuations.

We have clarified this rationale and its implications in the Introduction, Methods, and Results sections of the revised manuscript, emphasising how the frequency‑tagging approach mitigates potential motor confounds and enables the selective tracking of self‑, other‑, and integrative neural processes with high signal‑to‑noise ratio, despite the inherent motor components of such dynamic synchronisation tasks (p. 6, 25-26, 30, and 31).

*“Crucially, we combined this paradigm with dual‑EEG recordings and frequency‑tagging of the continuous auditory streams, enabling us to capture distinct neurophysiological markers of self- and other-related processing, as well as of their relative integration and segregation. This dual-brain tagging approach, previously applied to visually mediated interpersonal coordination (52), provides a unique opportunity to investigate the neural processes underlying behavioural coordination dynamics by disentangling sensory-related from motor-related activity – a key limitation of many current hyperscanning approaches (53-55).”*

*“The present findings highlight the strengths of combining dual-EEG and frequency tagging to investigate the fundamental behavioural and brain mechanisms that enable humans to interact through sound. This approach allowed us to record, with high signal-to-noise ratio, the neural processing of self- and other-produced sounds while preserving sufficient behavioural freedom to characterise the mechanisms participants used to achieve successful synchronisation…*

*…Importantly, this method departs from conventional hyperscanning approaches, which typically focus on correlational or synchrony‑based measures computed directly between the neural activities of interacting individuals (95-98). Such measures, primarily capturing temporal alignment across brains, make it difficult to dissociate cognitive and perceptual processes related to self–other distinctions from the behavioural synchrony and motor coupling that often accompany coordinated action (53-55). This limitation becomes especially pronounced in highly dynamic synchronisation contexts, such as those investigated in the present study, where motor alignment and shared sensory inputs are intrinsically confounded with higher‑level social and cognitive processes.*

*By contrast, dual‑EEG audio frequency tagging enables the selective isolation of self‑ and other‑related sensory and cognitive processes, independently of motor activity, thereby providing a more direct way to investigate the mechanisms underlying joint action. To our knowledge, this study provides the first demonstration that this new methodological approach can be leveraged to disentangle self–other processing during interpersonal coordination in the auditory domain. In doing so, it extends our previous work using dual-EEG frequency tagging in the visual modality (52), and suggests that that auditory interpersonal coordination relies on partially distinct processes.”*

*“Finally, the envelopes of the sounds produced by Person 1 and Person 2 were sinusoidally modulated (modulation index 1; (119)) at 33 Hz and 47 Hz, respectively, prior to binaural presentation. This frequency tagging approach enabled the separate recording, with high-signal-to-noise ratio, of neural responses specific to self- and other-generated sounds, and their integration, despite continuous changes in participants’ exerted force and corresponding changes in the acoustic signals (52, 64, 120).”*

*“Importantly, the 33 Hz and 47 Hz tagging signals were phase-locked across trials, enabling averaging prior to frequency-domain analyses to further enhance the signal-to-noise ratio of self-related, other-related, and intermodulation responses while attenuating non-phase-locked EEG activity (121).”*

5. The manuscript also lacks a description of hardware/software timing accuracy, timing alignment procedures, and drift correction - all essential components of hyperscanning methodology (e.g., see Zamm et al., 2024). Additional information on dual-EEG synchronization procedures is necessary.
We have now expanded the manuscript to provide a more detailed description of the hardware and software architecture, as well as the timing and synchronisation procedures used in the dual‑EEG setup.

The experimental system was fully controlled by a custom C++ application interfaced with Arduino hardware for real‑time acquisition of participants’ force input. This architecture was specifically designed to minimise latency and ensure precise, real‑time control of the generated auditory stimuli. Auditory signals were generated and presented at a sampling rate of 44 kHz, while motor inputs were updated at 60 Hz, resulting in a maximum system latency of approximately 17 ms.

All streams were centrally controlled within the same application, allowing precise temporal alignment and triggering of auditory stimulation and EEG recording. Synchronisation with the EEG data was ensured through shared triggers marking the onset of each trial. In addition, both participants’ EEG signals were recorded within the same BioSemi ActiveTwo system, ensuring a common time base and eliminating inter-system clock drift, thereby enabling accurate temporal alignment across participants.

We have clarified these aspects in the Methods section, including details on timing precision, synchronisation procedures, and system integration, to ensure transparency and to align with current best practices in hyperscanning research (p. 29 and 34).

*“The sound brightness of Person 1 and Person 2 was modulated in real-time for each pair based on the force that participants exerted with their right index finger on a force sensor (FlexiForce A401, Tekscan, Inc, MA, US). Force signals were recorded at 60 Hz using an Arduino Duemilanove board (Arduino, Ivrea, Italy) and processed by the C++ application to control online adjustments of sound brightness with minimal latency (17 ms maximum latency). The sound brightness was manipulated by linearly mapping the shape of the audio signal to the force exerted, with zero exerted force corresponding to a perfect sinusoidal waveform and maximum exerted force (> 111 N) corresponding to a perfect square waveform.”*

*“The Biosemi ActiveTwo system enabled simultaneous recording from both participants using a shared acquisition setup and integrated digital triggers marking the onset of each trial, ensuring precise temporal alignment of the EEG data across participants and within each experimental block.”*

B. Discussion: 
6. In the discussion, certain claims extend beyond what the data directly support. For example, EEG-based source localization, particularly with a template head model, has limited anatomical precision, especially for hemispheric lateralization. The authors should temper these anatomical claims and discuss the limitations of template-based source localization more explicitly.

We have revised the manuscript to temper the anatomical interpretations of the source localisation results and to more explicitly discuss the limitations inherent to EEG source localisation, particularly when using template (non‑individualised) head models. We now emphasise the need for confirmation using methods with higher spatial resolution in future research (p. 24-25).

*“While methods with higher spatial resolution, such as MEG with individual brain templates (88,89), will be needed to confirm this lateralisation in future research, stronger effects in the right hemisphere may be due to its superiority for processing spectral information, providing an advantage for self-other differentiation (39-40).”*

### While we acknowledge that EEG source localisation has limited anatomical precision, particularly at finer spatial scales, such limitations are less critical when interpreting broader spatial patterns, such as hemispheric differences. Indeed, hemispheric asymmetries are generally less prone to errors arising from template models, as they rely on coarser spatial distinctions (Michel & Brunet, 2019; Grech et al., 2008).

### In addition, the observed lateralisation is consistent with previous research on spectral processing and self‑related neural activity. We have therefore strengthened the Introduction and Discussion to better situate our findings within this existing literature, while ensuring that the interpretation remains appropriately cautious (p. 5 and 24-25).

*“Together, these findings suggest that distinct acoustic features and top-down attentional and predictive mechanisms may be especially important for self-other processing and joint synchronisation in natural environments characterised by temporally overlapping and less predictable sounds. Importantly, evidence suggests that the right hemisphere plays a prominent role in processing spectral and timbral sound features relevant for sound source differentiation (39,40), and also shows specialisation for self-related auditory representations and the sense of self more broadly (29,41-43). Related work also indicates that these mechanisms could be shaped by leadership roles during auditory interaction (15,44,45). While leader-follower roles are…”*

*“…stronger effects in the right hemisphere may be due to its superiority for processing spectral information, providing an advantage for self-other differentiation (39,40). Interestingly, converging evidence from previous studies also points to a right-hemisphere dominance for self-related processing, both in auditory self-representations and in the sense of self more generally (29,41-43). This is consistent with the stronger effects we observed for Self compared to Other, particularly in the right hemisphere, suggesting a critical role of the self-other distinction in maintaining control over one’s own actions and preventing agency confusion (90,91).”*

7. Interpretations involving participants' attentional expectations, "agency confusion," and hierarchical self-other processing along the auditory pathway should be presented more cautiously, as these mechanisms were not directly probed. Clearly distinguishing between empirically supported conclusions and more speculative theoretical interpretations would strengthen the discussion.

We have revised the Discussion to present these interpretations more cautiously throughout, particularly those related to attentional expectations, agency confusion, and hierarchical self–other processing. We have tempered these claims and clarified the distinction between empirically supported findings and more speculative theoretical interpretations. These revisions ensure that the conclusions remain closely aligned with the data while still highlighting their broader conceptual implications.

Reviewer #3: The study by Varlert et al. uses an auditory-motor synchronization paradigm to investigate how humans maintain a distinction between self- and other-generated sounds during joint action. The authors show that participants synchronize more effectively when their sounds are acoustically distinct and when leadership roles are assigned. I found the frequency-tagging approach to isolate neural responses to self- versus other-related stimuli particularly elegant, and the analyses and results are generally convincing. I have 3 major comments, as well as several smaller points, which I believe would strengthen the manuscript.

Major comments

1) Clarity of expression. The manuscript is very difficult to follow due to extremely long sentences and the use of technical terms before they are introduced or intuitively motivated. Early on, it is unclear what "inferring/coordinating from sound" refers to. The task description is especially hard to parse: even after repeated readings of the Fig. 1 caption, I struggled to understand (1) what the participant instructions actually were and (2) how exactly were the auditory signals experimentally manipulated. Several concepts appear abruptly (e.g., "brightness of sound", "frequency tagging"), though they are not widely familiar and require explanation for a general readership. In addition, some results are presented without even brief methodological context. Below, I point to a few particularly challenging passages; however, I would recommend that the manuscript be more broadly rewritten for clarity.

We thank the reviewer for this important comment. We agree that clarity of expression is critical, particularly given the technical nature of the study, and we have substantially revised the manuscript to improve readability throughout.

Specifically, we have clarified the overall structure and progression of the manuscript to better guide the reader from prior research to the aims of the study. Key concepts (e.g., sound brightness, frequency tagging, and interpersonal motor coordination) are now introduced earlier and explained more clearly to ensure accessibility for a broader readership. In addition, the task description has been thoroughly revised to make the participant instructions and the nature of the auditory manipulations more transparent.

Methodological details have also been expanded and reorganised. In particular, we have introduced a dedicated methodological section at the beginning of the Results section, ensuring that all key analytical procedures and measures are clearly described before the presentation of results. This allows readers to more easily interpret the reported findings.

Finally, the Results section has been revised to improve coherence between the description of observed patterns and their corresponding statistical analyses, so that each result is presented with its appropriate context.

We believe that these revisions substantially improve the clarity, readability, and accessibility of the manuscript.

2) Contextualization and theoretical framing. In my opinion, the title (phrases like "self-other distinction from sound") strongly suggests a link to voice or speech processing, yet this literature is not addressed at all. Once it becomes clear that the study is not about voice or speech, the reader might then expect connections to other more typical auditory-motor coordination paradigms - such as tapping, joint rhythmic entrainment, or musical synchronization - but these are also largely neglected. Instead, the introduction moves rapidly to a very specific and technically complex paradigm without providing intuitive conceptual grounding. I recommend introducing the motivation more directly, clarifying what kind of "coordination from sound" the study targets (and why), and situating the work more clearly within both the vocal-agency and sensorimotor-synchronization literatures so that readers can understand how this paradigm extends or complements existing (more classical) approaches.

We agree that clearer contextualisation and theoretical framing are essential, particularly given the technical nature of the paradigm.

In response, we have revised the Introduction to more directly introduce the overarching goal of the study and to clarify the specific form of auditory coordination being investigated. In particular, we now more explicitly situate our work within the broader literature on sensorimotor synchronisation, including joint rhythmic coordination and musical interaction, and we have strengthened the links to these established paradigms to provide more intuitive conceptual grounding. The present paradigm can be viewed as a natural extension of interpersonal sensorimotor synchronisation research, moving from discrete, rhythmically structured actions to continuous, dynamically evolving interactions.

In addition, we have expanded the discussion of relevant work on vocal and speech‑based interactions, which are directly relevant to self–other processing from sound. These literatures are now more clearly integrated into both the Introduction and Discussion, ensuring that the contribution of the present paradigm can be understood in relation to existing approaches.

Finally, we have revised the title and improved the overall flow of the Introduction to ensure that key concepts are introduced more gradually and that the reader is better guided from established frameworks to the specific contributions of the current study.

3) Results interpretation: top-down vs. bottom-up mechanisms. Several paragraphs in the introduction talk about hierarchical processes that might contribute to self-other distinction, but the argument remains difficult to follow, and the connection between these proposed top-down mechanisms and the present paradigm is not clearly articulated. For example, after the brief mention of "anticipation, adaptation, and ongoing planning" I was expecting that such processes would be manipulated or discussed, yet the manuscript never returns to them. In contrast, the broader auditory self-other literature often targets specific processes possibly guiding the hierarchical predictions: efference-copy mechanisms in self-voice monitoring (e.g., Whitford et al., 2019, Biological Psychiatry: CNN), memory-based self-voice representations (e.g., Iannotti et al., 2021, Cerebral Cortex), attentional and emotional biases (e.g., Pinheiro et al., 2022, Cortex), and even biases from concurrent sensory
stimuli such as bone conduction (e.g., Orepic et al., 2023, RSOS). These lines of research provide a clearer theoretical grounding for understanding how self-related auditory information is processed. Moreover, many of self-other studies also converge on right-hemisphere specialization for self-related processing - both the sense of self more broadly (Northoff et al., 2006, NeuroImage) but also within auditory self-representation (e.g., Candini et al., 2018, NeuroImage: Clinical; Iannotti et al., 2021, Cerebral Cortex; Kaplan et al., 2008, SCAN). I therefore recommend that the authors relate their own right-hemisphere findings to this wider self-other literature, both in the Introduction (for context) and more importantly in the Discussion.

We agree that the link between the proposed hierarchical mechanisms and the present paradigm required further clarification.

In response, we have revised the Introduction to more clearly articulate how the top‑down mechanisms discussed relate to the present experimental design (p. 4-5). In particular, we now explicitly describe how leadership roles serve as an experimental manipulation of top‑down influences on self‑ and other‑processing, thereby providing a more direct link between the theoretical framework and the empirical paradigm.

In addition, we have expanded the discussion of the auditory self–other literature, including work on speech and voice, which provides important insights into the mechanisms underlying self‑related auditory processing. This broader literature is now more clearly integrated into both the Introduction (p. 4-5) and Discussion (p. 27), helping to position the present work within established research on efference‑copy and related mechanisms supporting self–other distinction.

Furthermore, following the reviewer’s suggestion, we have incorporated relevant work on hemispheric specialisation, particularly the role of the right hemisphere in self‑related processing. These findings are now introduced earlier in the Introduction (p. 5) to provide additional context and are explicitly discussed in relation to our lateralisation results (p. 24-25), thereby strengthening the theoretical grounding of the study and situating our findings within the broader literature.

Overall, these revisions clarify the conceptual framework, strengthen the connection between theory and experiment, and provide a more comprehensive interpretation of the results.

Minor 

Line numbers would be helpful for the review

Line numbers have been included

Title & abstract
I find "action coordination through sound" quite vague
The first part of abstract is also rather unclear. It is hard to understand the scope of the study: are people speaking in synchrony or tapping in rhythm, or something else? I am also not convinced by the statement "such interaction is challenging in real-life". I'd argue that the brain does do this quite well in real life.
Other vague expressions: "leader-follower roles", "stronger facilitation", "modality-specific" (which modalities other than auditory are manipulated?)

The title has been revised to better reflect the scope and focus of the study.

The abstract has also been substantially revised to clarify the nature of the experimental paradigm and to make the scope of the research more explicit. In particular, we now more clearly describe the type of coordination task investigated, addressing potential ambiguities regarding whether the study involves speech, tapping, or other forms of interaction. We have also moderated the claim regarding the “challenging” nature of self–other separation.

In addition, previously vague expressions (e.g., leadership roles and “stronger facilitation”) have been clarified. The reference to “modality‑specific” effects has also been clarified in the manuscript, where it now explicitly refers to comparisons with previous work in the visual domain using a similar paradigm. For clarity and concision, this terminology has been removed from the abstract, where it could not be adequately elaborated.

Introduction

Page 4: 
"impossible in principle" - exclusive and too strong, not tested explicitly

Rephrased accordingly: *“can become particularly difficult”*

"growing evidence point to a complementary and necessary involvement of selective attentional and predictive mechanisms (…)" - such as? References?

"Such top-down brain mechanisms (…)" - my major comments

Revised to explain the mechanisms with reference to relevant literature (p. 4). Also see major comments above.

*“Second, a growing body of research shows that self-other differentiation in the auditory domain also relies on selective attentional and predictive mechanisms operating at higher levels of the auditory pathway (23-28). Top-down modulation of auditory processing has been linked to several complementary processes, including efference-copy-based attenuation of self-generated sounds, memory-based representations of self-related auditory input, and attentional biases toward behaviourally relevant sound sources (29-33). Related mechanisms have also been observed for other-generated sounds across a range of auditory interaction scenarios, from rhythmic synchronisation to speech processing, where attention and prediction contribute to the selective enhancement of relevant acoustic streams amid competing inputs (34-37). The internal simulation of others’ actions is thought to contribute to these effects, with forward models of observed behaviour potentially giving rise to efference‑copy–like predictions of expected sensory consequences that support anticipatory gain control and the differentiation of self‑ and other‑produced sounds (6,19,23,38).”*

Page 5:
"Here we combined dual-EEG and frequency-tagging techniques in a dyadic improvisation synchronization" - terms not clear for general readership.

The Introduction and Results sections have been revised to improve accessibility for a general audience, with clearer articulation of the motivations behind the approach and more detailed explanations of the technical aspects.

"an auditory-motor analogue of the mirror game paradigm" - please briefly mention what is the paradigm. The reader does not want to open another paper to properly understand this one

Similarly, the Introduction and Results sections have been revised to facilitate understanding of the paradigm, with a more explicit presentation of the underlying motivations and more detailed explanations of the experimental task and instructions.

"Illustration of the improvisation synchronisation task in which (…)" and "This frequency tagging of the sound envelope enabled neural processing (…)" - long sentences very hard to follow

Revised to use shorter sentences.

Fig 1: What instructions were given to participants? Upper panel - I find that the 2 rows are rather confusing than helping the interpretation - the only difference is the color, but then the color is introduced only below. The brightness aspect is not clear and not obvious if it is (and even should be) the same in both rows. Is this auditory manipulation evolving in time (the arrow)? Is this happening at each trial? Very hard to understand the figure without reading the methods.

The figure has been revised to improve clarity and facilitate interpretation. In particular, its structure has been reorganised to first present the joint auditory–motor synchronisation task, followed by the generation of the auditory signals, providing a more intuitive description. In addition, panel titles have been revised and supplementary annotations have been introduced to clarify key aspects of the experiment.

Together with the revisions made in the Introduction and Results sections—where further details on the experimental task, participant instructions, and auditory stimuli are now provided—these changes should improve overall comprehension, even without detailed reference to the Methods section.

"improvisation" suggests freedom, but "synchronisation" implies alignment. Not obvious how they are merged together in the paradigm.

The experimental task has now been clarified throughout the manuscript to better explain how “improvisation” and “synchronisation” are combined within the present paradigm. In particular, we emphasise that participants were required to generate self‑paced, dynamically evolving sound modulations (improvisation) while simultaneously aligning these fluctuations with those of their partner (synchronisation).

This combination is not unusual and closely relates to naturalistic contexts such as joint musical performance, where individuals produce flexible, creative actions while maintaining temporal and perceptual alignment with others. We have expanded the description of the task accordingly to make this dual requirement more explicit and intuitive for the reader (p. 5, 7, and 32).

*“We manipulated participants’ leadership roles and fundamental sound frequencies in a novel interpersonal improvisation paradigm in which pairs of participants synchronised self-paced fluctuations in continuous auditory streams presented binaurally and generated through ongoing fine‑grained force adjustments made with the index finger.”*

*“The paired participants were instructed to produce dynamic changes in sound brightness that were as original (i.e., in terms of temporal structure and magnitude) and as synchronised as possible, in line with similar “mirror game” experiments previously conducted in the visual domain (49,52). The two auditory streams were presented together to each participant binaurally via insert earphones. They were given practice trials to familiarise themselves both with the mapping between finger‑pressure changes and corresponding changes in sound, and with the synchronisation task.”*

*“Participants were then given instructions for the auditory-based improvisation synchronisation task. They were instructed to produce novel and dynamic modulations in sound brightness and to synchronise these modulations as closely as possible with those of their partner. Novelty could be achieved through continuous variations in both the magnitude and temporal structure of brightness changes, allowing participants to generate a wide range of distinct patterns over time. This was implemented in a self-paced manner, with no external metronome or timing cue, such that participants freely determined how to produce these dynamic changes while maintaining synchrony with their partner. This was followed by a brief practice phase in which both sounds were presented binaurally via the earphones, allowing participants to experience the combined auditory feedback.”*

The manuscript states that participants produced "original and synchronized fluctuations in brightness", but does not explain what these fluctuations should look like, whether they followed an external cue, or how participants knew they were on time with one another.

The description of the task has been clarified throughout the manuscript to better explain the nature of the fluctuations and how synchronisation was achieved.

In particular, participants generated self‑paced, continuously evolving fluctuations in sound brightness, without any external cue or metronome. This has now been clearly specified in the Introduction, Methods, and Results sections (p. 5, 7, and 32 – see above).

Synchronisation was achieved through shared auditory feedback: the sounds produced by both participants were combined in real time and presented binaurally via insert earphones. This allowed each participant to directly perceive both their own and their partner’s output, enabling them to adjust their actions online and maintain alignment. This aspect of the task has also been clarified throughout the revised manuscript.

The manipulation of brightness through finger pressure is highly unconventional and is not sufficiently explained in intuitive terms.
The manipulation of brightness through finger pressure is now explained more clearly and intuitively in both the Methods section and at the beginning of the Results section (p. 5, 7, and 32 – see above). These additions provide a more concrete description of the motor–auditory mapping and the task, which should make the paradigm easier to understand for a broad readership.

Why 33 and 47 Hz exactly? Isn't it critical to mention that these amplitude fluctuations cannot be consciously perceived?

The choice of tagging frequencies (33 and 47 Hz) was guided by methodological considerations, as these frequencies are known to elicit robust and reliable auditory steady‑state EEG responses.

In addition, they provide a suitable balance between clear separability of self‑ and other‑related signals in the frequency domain and the minimisation of distortion that may arise at both lower and higher frequencies. These points have now been clarified in the revised manuscript in both the Results (p. 8) and Methods (p. 31) sections.

*“The tagging frequencies were selected due to their proximity to 40 Hz, which is known to elicit robust auditory steady‑state EEG responses (65,66), while remaining sufficiently separated to allow clear dissociation of self‑, other‑, and intermodulation‑related signals, and high enough to minimise contamination from low‑frequency neural activity and movement‑related artefacts (67).”*

*“The modulation frequencies were selected because they reliably elicit auditory steady-state EEG responses while being sufficiently separated in the frequency domain to allow unambiguous dissociation of self-related, other-related, and intermodulation components, and to minimise overlap with low-frequency neural oscillations and movement-related artefacts (67). In addition, choosing modulation frequencies below 50 Hz ensured that the resulting higher-order intermodulation frequency (80 Hz) remained well below 100 Hz, thereby avoiding higher frequency ranges where EEG signal-to-noise ratios are reduced (121).”*

Regarding perceptibility, although amplitude modulations at these frequencies are generally subtle, it is possible that they remained perceptible. Importantly, this would not critically affect the present findings. The experimental design relies on within-subject comparisons of behavioural and neural responses across conditions. Therefore, any degree of conscious perception of the tagging frequencies would be expected to affect all conditions equivalently and would not introduce systematic bias between them.

Intermodulation frequencies - not obvious why they would indicate integration. Does their processing necessarily reflects integration? Could low-level auditory processing be really decoupled from higher-level mechanisms (e.g. paying attention to self/other identity)?

Intermodulation frequencies arise from nonlinear interactions between concurrently presented frequency‑tagged signals. Their presence indicates that information carried by the two tagged streams is processed within overlapping or interacting neural populations (p. 8 and 31), but does not, by itself, specify the level of processing at which this interaction occurs. Accordingly, these responses are best interpreted as reflecting joint processing, with the precise level of integration—from low‑level to higher‑level mechanisms—remaining to be determined, which is why we adopt a cautious interpretation.

In this respect, the modelling analyses provided in the supplementary material are informative, suggesting that the interaction between the signals may occur at relatively early stages of the auditory pathway. Importantly, we do not assume that self–other integration is necessarily independent from the processing of the individual streams. However, our results do indicate some degree of dissociation, which is now explicitly discussed in the revised manuscript (p. 25-26).

*“Crucially, this method not only enables the tracking of self- and other-specific processes, but also provides a direct index of their degree of integration, capturing a range of interconnected yet partially independent mechanisms along the auditory pathway (39,92-94). For instance, leadership-based selective neural enhancement of self- and other-related information did not appear to extend to their integration. Additionally, the reduction in intermodulation-related responses associated with distinct fundamental sound frequencies was accompanied, not by a decrease but by an actual increase in self-related responses, highlighting the dissociation between self-, other-, and integrative processing.”*

Results

Coherence - briefly mention how it is computed

We have added further details (p. 9), as recommended.

*“To assess the degree of synchronisation between the dynamic modulations of the two participants’ continuous tones across conditions, we calculated the cross-spectral coherence between their signals (68,69). Coherence was computed as the normalised cross-spectrum of the two time series, obtained by dividing the magnitude of their cross-power spectral density by the product of their individual power densities. This measure captures complex alignment emerging across a range of timescales, as illustrated in Fig 2.”*

ANOVAs - was there an interaction term and why yes or why not (in all analyses)?

Yes, interaction terms were included in all ANOVA models. Overall, there was no evidence for meaningful interactions between Leadership and Sound Frequency across the variables, with only a marginal or isolated effect that did not yield a clear or interpretable pattern. In the revised manuscript, all interaction effects are now explicitly reported, together with their corresponding test statistics, p‑values, effect sizes, as well as the means and confidence intervals for all conditions.

Fig 2 - "1 × 95% CI of the mean computed for within-subject designs" - not clear

This description was previously missing and has now been added to the statistical analysis section of the Methods. We now explicitly clarify how the confidence intervals are computed for within‑subject designs and how they relate to the interpretation of the effects (p. 37-38). In particular, these intervals reflect within‑participant variability and therefore provide an appropriate estimate of uncertainty around condition differences in repeated‑measures analyses.

*“Confidence intervals presented in the text and figures (as error bars) are computed as within‑subject 95% confidence following the method described by Morey (2008) (72), which appropriately accounts for the dependency structure inherent in repeated‑measures designs. This approach ensures that the confidence intervals reflect within‑participant variability rather than between‑participant differences, providing a more accurate representation of condition effects.”*

Why up to 5 Hz?

The upper frequency limit (5 Hz) was selected to correspond to the typical range of human movement dynamics and interpersonal synchronisation processes. This choice ensures that the analyses capture behaviourally relevant fluctuations in the signals. This rationale is now explicitly clarified in the revised Results section (p. 9).

*“This measure captures complex alignment emerging across a range of timescales, as illustrated in Fig 2. The analysis was restricted to frequencies up to 5 Hz, encompassing the range typically associated with human rhythmic movement production and synchronisation (70,71).”*

Page 9: Why is Leadership variable now modeled differently (3 levels instead of 2)? Why is this not consistent across analyses (or both reported for all)?

The difference in the modelling of the Leadership factor reflects the level of analysis rather than an inconsistency. At the dyad level, Leadership is treated as a two‑level factor (Designated, Undesignated), whereas at the individual level it is represented with three levels (Leader, Follower, Joint) to capture participant‑specific roles. This distinction has now been clarified in both the Results (p. 10) and Methods (p. 36-37) sections.

*“A repeated-measures ANOVA with the factors Leadership (Designated [average of leader-follower and follower-leader conditions] and Undesignated [joint condition]) and Sound Frequency (Matched and Unmatched) conducted on the average coherence values yielded significant main effects of Leadership, F(1, 24) = 10.67, p = 0.003, η_g_^2^ = 0.06, and Sound Frequency, F(1, 24) = 36.99, p < 0.0001, η_g_^2^ = 0.04.”*

*“Conditions with identical sound-frequency pairings (200 Hz – 200 Hz and 500 Hz – 500 Hz) and with different sound-frequency pairings (200 Hz – 500 Hz and 200 Hz – 500 Hz) were averaged to form two Sound Frequency conditions – Matched and Unmatched. Two-way repeated-measures ANOVAs with the factors Leadership (Leader, Follower, and Joint) and Sound Frequency (Matched and Unmatched) were then conducted on participants’ mean sound brightness, and EEG amplitudes at the Self (33 Hz and 47 Hz for Person 1 and Person 2, respectively), Other (47 Hz and 33 Hz for Person 1 and Person 2, respectively), and 14 Hz and 80 Hz Inter-modulation frequencies. Individual-level conditions in these analyses were thus based on a total of 12 minutes of continuous interaction, providing reliable behavioural and neural estimates.*

*In contrast, interpersonal behavioural coherence was analysed at the dyadic level. Leader-Follower and Follower-Leader conditions were therefore averaged to form two Leadership conditions – Designated and Undesignated – and analysed using a two-way repeated-measures ANOVA with the factors Leadership (Designated and Undesignated) and Sound Frequency (Matched and Unmatched). This averaging reflects the inherently dyadic nature of the coherence measure, with each resulting condition represented by a total of at least 12 minutes of continuous interaction, enabling reliable estimation of interpersonal synchronisation.”*

Fig 3 - it would be helpful to indicate what is significant. The same for some other plots.
Significant effects are now indicated where applicable in the figures, including Figures 2, 3, 6, and 8.

Why averaging across all EEG channels?

Averaging across EEG channels was done to increase the signal‑to‑noise ratio and enhance the detection of frequency‑tagged responses at the scalp level, in line with previous research, by reinforcing consistent signal components while reducing noise that varies across electrodes. This approach allows for the detection of effects irrespective of their precise spatial origins, while topographic and source‑space analyses are used to visualise where these effects are strongest. This point has now been clarified in the revised manuscript (p. 35).

*“This averaging reduces dimensionality and increases the signal‑to‑noise ratio by reinforcing responses that are consistent across channels (52,75).”*

Fig 4 - What is the take-home message of this plot? Left: There are a few other prominent peaks than the 4 indicated ones. What could they represent? Is it then justified to only use 14 and 80 for further analyses? Can there be other markers of integration (e.g. some other nonlinear combination of 33 and 47)? Right: The 80 peak does not seem to stand out from many others. Can it therefore be used reliably in further analyses?
The figure illustrates the clear spectral peaks obtained at the tagging frequencies and their corresponding intermodulation frequencies, highlighting the high signal‑to‑noise ratio achieved with the dual‑EEG frequency‑tagging approach. Additional peaks visible in the spectrum correspond to higher harmonics and other nonlinear components, which are typically of lower magnitude and not consistently observed across participants; for this reason, they were not included in further analyses. In the revised manuscript, we have added further explanations and individual statistical tests at the relevant frequencies to assess the consistency of the selected responses across participants (p. 12-13).

*“Fig 4 presents baseline-subtracted frequency-domain amplitude spectra averaged across all conditions and dyads, together with the corresponding scalp topographies. Clear responses were observed at the tagging frequencies corresponding to the participant’s own sound (Self), their partner’s sound (Other), and their integration, as reflected in significant group z-score values at 33 Hz, 47 Hz, and 14 Hz and 80 Hz (i.e., 47 – 33 and 33 + 47) – see Materials and methods for further details about significance testing. The corresponding scalp distributions were consistent with auditory cortical activity, as typically observed with an average-reference montage (74,75).*

*Reliable responses at these frequencies were observed consistently across participants (significant z-score values for all participants for Self and Other, and 70% and 32% of the participants for 14 Hz and 80 Hz, respectively). Reponses at the second harmonic of each frequency were of markedly lower amplitude and were generally less reliably present across participants (48%, 50%, 18%, and 32% of the participant sample with significant z-score values, respectively); consequently, they were not retained for further analyses.”*

Regarding the 80 Hz response, we agree that it is less reliably observed, and its interpretation has therefore been revised and is now discussed more cautiously in the manuscript (p. 19). However, it should be noted that the overall pattern of results remains broadly consistent with the 14 Hz intermodulation response.

*“Source-space analyses confirmed this effect, showing a stronger right-hemispheric involvement for the 14 Hz response, whereas no clear hemispheric asymmetry was observed at 80 Hz. While caution is warranted given that 80 Hz responses were generally less reliable, these results suggest that the effect of distinct sound frequencies was generally more bilaterally distributed for the intermodulation frequencies than for self and other frequencies”*

"suggesting an increase in the neural tracking (…) not only originating from peripheral changes in sound brightness but also centrally from selective enhancement at the brain level" Do we even know that there are any peripheral changes (I assume here you mean the cochlea or auditory nerve?) Not sure this statement is justified if this was not manipulated.

We agree that this statement required clarification. The reference to “peripheral changes” does not imply direct measurement of cochlear or auditory‑nerve activity, but instead reflects changes in the acoustic signal itself associated with variations in sound brightness. These differences can lead to increased EEG response amplitudes, reflecting stronger neural tracking of the stimulus. We have revised the manuscript to make explicit that this interpretation refers to stimulus‑driven acoustic changes rather than direct measurement of peripheral mechanisms.

Importantly, beyond these stimulus‑driven effects, our results also indicate condition‑dependent modulations that cannot be fully explained by brightness differences alone, supporting the contribution of central, task‑related processes. This distinction has now been clarified in the revised manuscript (p. 14-15).

*“This dissociation suggests that the enhancement of neural responses to self-produced sounds cannot be fully explained by peripheral, stimulus-driven differences in acoustic input. Instead, it points to an additional contribution from centrally mediated mechanisms, consistent with selective enhancement of self-related auditory signals at the cortical level as a function of leadership roles.*

*To directly test whether leadership-related differences in Self responses could be explained by differences in sound brightness alone, we conducted multiple linear regression analyses. These analyses allowed us to statistically control for the contribution of peripheral acoustic variation while assessing the independent predictive value of leadership roles (Fig 5). This analysis showed that participants’ own sound brightness was a significant predictor of EEG Self response amplitude, t(146) = 4.97, p < 0.0001. Importantly, however, leadership roles also explained additional variance beyond sound brightness, with both Joint, t(146) = 3.36, p = 0.001, and Leader, t(146) = 2.73, p = 0.007, being significant predictors. Consistent with this interpretation, a model including the factor Leadership, F(3, 146) = 23.85, p < 0.0001, R^2^ = 0.32, explained significantly more variance, F(2, 146) = 6.30, p = 0.002, than a simpler model including only Self sound brightness, F(1, 148) = 55.02, p < 0.0001, R^2^ = 0.27.*

*Together, these regression results further indicate that leadership-related modulations of neural responses to self-produced sounds cannot be reduced to peripheral acoustic differences alone. Rather, they suggest that participants’ interactive roles contributed independently to the cortical weighting of self-generated auditory input, supporting the interpretation of a role-dependent, top‑down modulation of self-related auditory processing.”*

Fig 5 - what are the units on the x and y axes?

The units have been added on all figures.

"These analyses also show that it is the right hemisphere that benefits the most from designated leadership roles, which could reflect the spectral superiority of this hemisphere" What is "spectral superiority of a brain hemisphere"?

This has been rephrased (p. 15-16).

*“These analyses also show that it is the right hemisphere that benefits the most from designated leadership roles, in line with greater sensitivity of this hemisphere to the spectral features of sounds and its involvement in self-other processing.”*

Fig 6 - Could the response be modulated by the absolute value of the 2 chosen frequencies? Why the choice of 200 and 500?

The response could in principle be influenced by the absolute values of the carrier frequencies. The choice of 200 Hz and 500 Hz was guided by methodological and perceptual considerations, including discriminability and robustness of the auditory signal. This rationale is now clarified at the beginning of the Results section (p. 7) and in the Methods section (p. 30).

*“The participants’ tones had either matched or unmatched fundamental frequencies (i.e., 200 and 500 Hz), and participants were either given distinct leader and follower roles (leader-follower and follower-leader conditions) or no leadership roles (joint condition) (see Fig 1). These frequencies were selected because (i) they fall within an ecologically valid range for human vocal communication, including singing (57,58), and (ii) are low enough to elicit robust EEG responses and perceptual differences in sound brightness, while remaining clearly distinguishable (59,60).”*

*“The fundamental sound frequency of Person 1 and Person 2, which remained constant throughout each trial, was either 200 Hz or 500 Hz, depending on the experimental condition. These frequencies (200 Hz and 500 Hz) were selected for both methodological and ecological reasons. Low carrier frequencies are known to elicit robust, high signal‑to‑noise ratio EEG responses, while remaining within an ecologically valid range for vocal sounds (57-60). Using relatively low carrier frequencies was also important for effective perceptual manipulation of sound brightness, as changes in waveform shape from sinusoidal to square waves yield salient timbral differences at low fundamental frequencies, whereas these differences become less perceptually distinct at higher frequencies, likely due to a coarser auditory encoding of the spectral envelope and increased interaction between pitch and brightness cues (117,118). The spacing between the two carrier frequencies therefore reflected a compromise between perceptual discriminability and neurophysiological sensitivity, with frequencies sufficiently separated to be easily distinguishable while remaining low enough to preserve strong EEG response amplitudes and reliably perceived sound brightness modulations.”*

What about the effect of Leadership in the "Self-Other neural integration/segregation" part? Do you observe similar effects for other prominent peaks from Fig 4?

No consistent effects of Leadership were observed in the self–other neural integration/segregation measures. All non‑significant effects are now explicitly reported in the revised manuscript, including the corresponding test statistics, p‑values, effect sizes, as well as the means and confidence intervals for the different conditions.

Regarding other peaks visible in Figure 4, no additional analyses were conducted, as these components were of lower magnitude and not consistently observed across participants. The analyses were therefore focused on the 14 Hz and 80 Hz intermodulation frequencies, which were more robust at the group level. Individual‑level statistical tests have been included to demonstrate the consistency of these selected responses across participants.

The dual-EEG setup is interesting, but only within-participant analyses are reported. Have the authors explored inter-brain synchrony or leader-follower dynamics? Measures such as cross-brain coherence or correlation of frequency-tagged responses could reveal how neural coordination supports behavioral synchrony. Such findings would largely strengthen the manuscript.

Inter‑brain synchrony measures were not the focus of the present study. In highly dynamic interaction paradigms involving continuous motor activity, such measures can be difficult to interpret, as shared motor behaviour can strongly drive apparent coherence or correlation between brains, independently of the underlying cognitive or sensory processes of interest.

This limitation is precisely one of the motivations for the dual‑EEG frequency‑tagging approach used here, which enables the selective isolation of stimulus‑locked neural responses and helps dissociate sensory processing of self‑ and other‑generated signals from motor‑related activity that may confound traditional hyperscanning measures. This advantage of the approach has now been more clearly articulated in the Introduction and Discussion sections (p. 6 and 26).

While cross‑brain correlation of frequency‑tagged responses could represent an interesting direction, such analyses would likely remain influenced by shared motor dynamics and signal‑to‑noise constraints, and would require further methodological development to enable unambiguous interpretation. We therefore consider this avenue to be beyond the scope of the current study, but an important direction for future research.

*“Crucially, we combined this paradigm with dual‑EEG recordings and frequency‑tagging of the continuous auditory streams, enabling us to capture distinct neurophysiological markers of self- and other-related processing, as well as of their relative integration and segregation. This dual-brain tagging approach, previously applied to visually mediated interpersonal coordination (52), provides a unique opportunity to investigate the neural processes underlying behavioural coordination dynamics by disentangling sensory-related from motor-related activity – a key limitation of many current hyperscanning approaches (53-55).”*

*“Importantly, this method departs from conventional hyperscanning approaches, which typically focus on correlational or synchrony‑based measures computed directly between the neural activities of interacting individuals (95-98). Such measures, primarily capturing temporal alignment across brains, make it difficult to dissociate cognitive and perceptual processes related to self–other distinctions from the behavioural synchrony and motor coupling that often accompany coordinated action (53-55). This limitation becomes especially pronounced in highly dynamic synchronisation contexts, such as those investigated in the present study, where motor alignment and shared sensory inputs are intrinsically confounded with higher‑level social and cognitive processes.*

*By contrast, dual‑EEG audio frequency tagging enables the selective isolation of self‑ and other‑related sensory and cognitive processes, independently of motor activity, thereby providing a more direct way to investigate the mechanisms underlying joint action. To our knowledge, this study provides the first demonstration that this new methodological approach can be leveraged to disentangle self–other processing during interpersonal coordination in the auditory domain.”*

Discussion
"Our work (…) demonstrates that the processes underlying interpersonal coordination are modality-specific." - How does this study show that? What modality other than auditory is tested?

The reference to modality‑specific processes is based on comparisons with our previous work using a closely related paradigm in the visual domain. While the present study focuses on auditory interaction, the differences observed relative to this prior work suggest that the mechanisms supporting interpersonal coordination may partly depend on the sensory modality.

This comparison has now been clarified in the revised manuscript, particularly in the Discussion and Conclusion, where we explicitly situate the present findings in relation to the visual paradigm and interpret the modality‑specific aspect more cautiously (p. 26-27 and 28).

*“To our knowledge, this study provides the first demonstration that this new methodological approach can be leveraged to disentangle self–other processing during interpersonal coordination in the auditory domain. In doing so, it extends our previous work using dual-EEG frequency tagging in the visual modality (52), and suggests that that auditory interpersonal coordination relies on partially distinct processes.*

*Notably, the central role of the self-other distinction observed here contrasts with findings observed by Varlet et al. (2020) (52) using a similar synchronisation paradigm in the visual domain, where self-other integration, facilitated by movement similarity and undesignated leadership roles, was prioritised to improve interpersonal coordination. While future research directly comparing modalities will be needed to confirm and generalise this pattern, the present results suggest that self-other integration and segregation may be uniquely constrained and balanced across sensory modalities (99-101). Such modality-specific constraints may influence how information exchange and action coordination are established and maintained between individuals (14).”*

*“Such mechanisms may help humans compensate for self-other masking that can occur with acoustic signals, and may therefore differ from those previously observed in visually-guided interactions, where coordination appears to rely more strongly on self-other integration through action and movement similarity.”*

Broader scope - why this work is important - could be strengthened. Specifically, there is a large body of literature demonstrating that auditory self-other confusion is related to auditory-verbal hallucinations, both in schizophrenia and in healthy individuals (e.g. Duggirala et al., 2024, Schizophrenia Bulletin; Waters et al., 2012, Schizophrenia Bulletin) and in other clinical changes in the sense of self (e.g. Voruz et al., 2024, Heliyon; Candini et al., 2018, NeuroImage: Clinical)

We agree that the broader implications of the work could be strengthened. In response, we have expanded the Discussion to better situate our findings within the wider literature on auditory self–other distinction, particularly in relation to clinical and subclinical phenomena such as auditory‑verbal hallucinations and alterations in the sense of self.

In particular, we have incorporated the suggested references and clarified how our framework relates to existing work linking auditory self–other confusion to schizophrenia and related conditions, as well as to more general mechanisms of self‑representation. These additions strengthen the broader relevance of the study and highlight its potential implications for understanding disruptions in self–other processing (p. 27).

*“This framework also offers new opportunities to investigate the interactional and communicative impairments associated with mental disorders and related conditions. It has direct relevance to understand interpersonal coordination disorders reported in patients with autism, schizophrenia, and social phobia (108-113). More generally, a substantial body of evidence indicates that atypical auditory self–other processing is closely associated with auditory‑verbal hallucinations in schizophrenia and subclinical populations, as well as with broader disruptions of the sense of self across a range of clinical conditions (41, 114-116). The present framework therefore opens new avenues for examining the mechanisms affected in these populations…”*

Methods

Sill not clear to me what exactly the instruction for the participants were. How was brightness of sound explained? How exactly is one told to lead?

The instructions provided to participants have now been clarified in both the Methods and Results sections (p. 7 and 32-33). In particular, we provide a more explicit and intuitive description of sound brightness, clearly explain how participants were instructed to generate and synchronise these fluctuations, and detail how leadership was operationalised during the task.

*“The paired participants were instructed to produce dynamic changes in sound brightness that were as original (i.e., in terms of temporal structure and magnitude) and as synchronised as possible, in line with similar “mirror game” experiments previously conducted in the visual domain (49,52). The two auditory streams were presented together to each participant binaurally via insert earphones.”*

*“Participants were then given instructions for the auditory-based improvisation synchronisation task. They were instructed to produce novel and dynamic modulations in sound brightness and to synchronise these modulations as closely as possible with those of their partner. Novelty could be achieved through continuous variations in both the magnitude and temporal structure of brightness changes, allowing participants to generate a wide range of distinct patterns over time. This was implemented in a self-paced manner, with no external metronome or timing cue, such that participants freely determined how to produce these dynamic changes while maintaining synchrony with their partner. This was followed by a brief practice phase in which both sounds were presented binaurally via the earphones, allowing participants to experience the combined auditory feedback. Participants then completed two practice trials in randomly selected experimental conditions before beginning the main task.*

*The participants were instructed to perform the task under three different Leadership conditions, as in the Varlet et al. (2020) (52) visual coordination study – the Leader-Follower condition, in which Person 1 was instructed to lead the improvisation; the Follower-Leader condition, in which Person 2 was instructed to lead the improvisation; and the Joint condition, in which no leader and follower roles were designated, and participants were instructed to perform together. In the conditions with a leader, the designated leader was instructed to initiate and shape the temporal dynamics of the sound modulations, while the other participant adapted their behaviour to maintain synchronisation.”*

References:

Gell, M., Eickhoff, S. B., Omidvarnia, A., Küppers, V., Patil, K. R., Satterthwaite, T. D., ... & Langner, R. (2024). How measurement noise limits the accuracy of brain-behaviour predictions. *Nature Communications*, *15*(1), 10678.

Grech, R. et al. (2008). Review on solving the inverse problem in EEG source analysis. Journal of NeuroEngineering and Rehabilitation, 5, 25.

Le Guern, N., Allouch, S., Pascucci, D., Ebadi, A., & Hassan, M. (2026). Robust EEG brain-behavior associations emerge only at large sample sizes. *bioRxiv*, 2026-02.

Michel, C. M., & Brunet, D. (2019). EEG source imaging: A practical review. Brain Topography, 32, 143–157.

Norcia, A. M., Appelbaum, L. G., Ales, J. M., Cottereau, B. R., & Rossion, B. (2015). The steady-state visual evoked potential in vision research: A review. Journal of vision, 15(6), 4-4.

Schubert, E., & Wolfe, J. (2006). Does timbral brightness scale with frequency and spectral centroid. *Acta Acustica*, *92*, 820-825.

Wallmark, Z., Nghiem, L., & Marks, L. E. (2021). Does timbre modulate visual perception? Exploring crossmodal interactions. *Music Perception*, *39*(1), 1-20.
