# Supplementary material for: Lateralised neural mechanisms facilitate auditory self-other distinction and coordination in human dyads
Source: PLoS Biol. 2026 Aug 10;24(8):e3003935. doi: 10.1371/journal.pbio.3003935 (PMC13475979; doi:10.1371/journal.pbio.3003935)
Supplement: Peer review history

# Editor report: Decision Letter 0

**DOI:** 10.1371/journal.pbio.3003935.r001

**Authors:** Christian Schnell, PhD

3 Oct 2025

Dear Dr Varlet,

Thank you for submitting your manuscript entitled "Who is who? Behavioural and brain mechanisms underpinning the self-other distinction and action coordination through sound" for consideration as a Research Article by PLOS Biology.

Your manuscript has now been evaluated by the PLOS Biology editorial staff and I am writing to let you know that we would like to send your submission out for external peer review.

However, before we can send your manuscript to reviewers, we need you to complete your submission by providing the metadata that is required for full assessment. To this end, please login to Editorial Manager where you will find the paper in the 'Submissions Needing Revisions' folder on your homepage. Please click 'Revise Submission' from the Action Links and complete all additional questions in the submission questionnaire.

Once your full submission is complete, your paper will undergo a series of checks in preparation for peer review. After your manuscript has passed the checks it will be sent out for review. To provide the metadata for your submission, please Login to Editorial Manager (https://www.editorialmanager.com/pbiology) within two working days, i.e. by Oct 07 2025 11:59PM.

If your manuscript has been previously peer-reviewed at another journal, PLOS Biology is willing to work with those reviews in order to avoid re-starting the process. Submission of the previous reviews is entirely optional and our ability to use them effectively will depend on the willingness of the previous journal to confirm the content of the reports and share the reviewer identities. Please note that we reserve the right to invite additional reviewers if we consider that additional/independent reviewers are needed, although we aim to avoid this as far as possible. In our experience, working with previous reviews does save time.

If you would like us to consider previous reviewer reports, please edit your cover letter to let us know and include the name of the journal where the work was previously considered and the manuscript ID it was given. In addition, please upload a response to the reviews as a 'Prior Peer Review' file type, which should include the reports in full and a point-by-point reply detailing how you have or plan to address the reviewers' concerns.

During the process of completing your manuscript submission, you will be invited to opt-in to posting your pre-review manuscript as a bioRxiv preprint. Visit http://journals.plos.org/plosbiology/s/preprints for full details. If you consent to posting your current manuscript as a preprint, please upload a single Preprint PDF.

Feel free to email us at plosbiology@plos.org if you have any queries relating to your submission.

Kind regards,

Christian

Christian Schnell, PhD

Senior Editor

PLOS Biology

cschnell@plos.org

# Peer review: Decision Letter 1

**DOI:** 10.1371/journal.pbio.3003935.r002

**Authors:** Christian Schnell, PhD

1 Dec 2025

Dear Dr Varlet,

Thank you for your patience while your manuscript "Who is who? Behavioural and brain mechanisms underpinning the self-other distinction and action coordination through sound" was peer-reviewed at PLOS Biology. It has now been evaluated by the PLOS Biology editors, an Academic Editor with relevant expertise, and by several independent reviewers.

In light of the reviews, which you will find at the end of this email, we would like to invite you to revise the work to thoroughly address the reviewers' reports.

As you will see below, the reviewers overall think that your study is well executed and provides important insights. However, they also raised a number of concerns about a lack of clarity, the statistical analyses and some potential confounders, including the small number of trials in each condition (Reviewer 2, point 2).

Given the extent of revision needed, we cannot make a decision about publication until we have seen the revised manuscript and your response to the reviewers' comments. Your revised manuscript is likely to be sent for further evaluation by all or a subset of the reviewers.

In addition to these revisions, you will need to complete some formatting changes, which you will receive in a follow up email. A member of our team will be in touch with a set of requests shortly.

We expect to receive your revised manuscript within 3 months. Please email us (plosbiology@plos.org) if you have any questions or concerns, or would like to request an extension.

At this stage, your manuscript remains formally under active consideration at our journal; please notify us by email if you do not intend to submit a revision so that we may withdraw it.

**IMPORTANT - SUBMITTING YOUR REVISION**

Your revisions should address the specific points made by each reviewer. Please submit the following files along with your revised manuscript:

1. A 'Response to Reviewers' file - this should detail your responses to the editorial requests, present a point-by-point response to all of the reviewers' comments, and indicate the changes made to the manuscript.

*NOTE: In your point-by-point response to the reviewers, please provide the full context of each review. Do not selectively quote paragraphs or sentences to reply to. The entire set of reviewer comments should be present in full and each specific point should be responded to individually, point by point.

You should also cite any additional relevant literature that has been published since the original submission and mention any additional citations in your response.

2. In addition to a clean copy of the manuscript, please also upload a 'track-changes' version of your manuscript that specifies the edits made. This should be uploaded as a "Revised Article with Changes Highlighted" file type.

*Re-submission Checklist*

When you are ready to resubmit your revised manuscript, please refer to this re-submission checklist: https://plos.io/Biology_Checklist

To submit a revised version of your manuscript, please go to https://www.editorialmanager.com/pbiology/ and log in as an Author. Click the link labelled 'Submissions Needing Revision' where you will find your submission record.

Please make sure to read the following important policies and guidelines while preparing your revision:

*Published Peer Review*

Please note while forming your response, if your article is accepted, you may have the opportunity to make the peer review history publicly available. The record will include editor decision letters (with reviews) and your responses to reviewer comments. If eligible, we will contact you to opt in or out. Please see here for more details:

https://blogs.plos.org/plos/2019/05/plos-journals-now-open-for-published-peer-review/

*PLOS Data Policy*

Please note that as a condition of publication PLOS' data policy (http://journals.plos.org/plosbiology/s/data-availability) requires that you make available all data used to draw the conclusions arrived at in your manuscript. If you have not already done so, you must include any data used in your manuscript either in appropriate repositories, within the body of the manuscript, or as supporting information (N.B. this includes any numerical values that were used to generate graphs, histograms etc.). For an example see here: http://www.plosbiology.org/article/info%3Adoi%2F10.1371%2Fjournal.pbio.1001908#s5

*Protocols deposition*

To enhance the reproducibility of your results, we recommend that if applicable you deposit your laboratory protocols in protocols.io, where a protocol can be assigned its own identifier (DOI) such that it can be cited independently in the future. Additionally, PLOS ONE offers an option for publishing peer-reviewed Lab Protocol articles, which describe protocols hosted on protocols.io. Read more information on sharing protocols at https://plos.org/protocols?utm_medium=editorial-email&utm_source=authorletters&utm_campaign=protocols

Thank you again for your submission to our journal. We hope that our editorial process has been constructive thus far, and we welcome your feedback at any time. Please don't hesitate to contact us if you have any questions or comments.

Sincerely,

Christian

Christian Schnell, PhD,

Senior Editor

PLOS Biology

cschnell@plos.org

------------------------------------

REVIEWS:

Reviewer #1: Thank you for the opportunity to read this very interesting and timely paper. I found it very well written and a valuable addition to research on social neuroscience of coordination. The findings have implications for broader work on communication and validate a novel paradigm that could be adapted to test these more ecologically-valid, complex interaction contexts. I have relatively minor comments below that I hope will add to the clarity of the manuscript and broaden its reach to multi-disciplinary audiences.

Introduction:

- It would be helpful for the reader to be more clearly guided through the Introduction, adding clear link from previous research to the focus of the current study. Can the research question be brought in earlier to help frame the background? The main study hypothesis is also presented in a long sentence of dense language- this would benefit from being split into two sentences with more concrete language: "We hypothesised that these mechanisms, together with those based on lower-level acoustic differences, constitute a powerful and flexible processing gradient enabling stream segregation at peripheral and central levels along the auditory pathway, key to maintaining the self-other distinction in everyday dynamic interactions characterised by temporally overlapping acoustic signals and uncertainty."

- The definition for 'brightness'- the key manipulation for participants in this study is buried in the Figure 1 legend - please define this key term in the main text.

Results:

- The pattern is results is described before (and independent of) stats, which makes hard to match up.

- Some effects are not mentioned at all (eg. leadership x frequency on interpersonal synchronisation, or main effect and interaction on sound brightness)- I assume these were all non-significant, but at the very least they should be acknowledged as such in the text with maximal p-value.

- Plots only show significant effects, rather than means for the fully crossed designs- non-significant factors are excluded. Please show complete plots of fully crossed data for transparency.

- Analysis of neural tracking- it's not clear why regressions are needed, they are presented as simply 'confirming' the ANOVA results?

- Currently behavioural and neural data are considered/analysed completed separately. Can additional analyses be run to test for links between these datasets, to support interpretation of the oscillations data?

Method:

- Please justify your sample size and comment on power. Did any of the dyads know each other before the experiment?

Reviewer #2: This innovative study combined dual-EEG and frequency-tagging techniques in a more ecological dyadic improvisation synchronization task to investigate a relevant and relatively understudied question in auditory perception: how individuals discriminate between self- and other-generated sounds in joint auditory-motor synchronization. By integrating behavioral and neural measures, the study shows that auditory self-other distinction is modulated by both stimulus characteristics (spectral information) and participant's control over one's own actions (leader-follower roles). These findings have important implications for our understanding of the mechanisms underlying social coordination in auditory interactions. There are several laudatory aspects to highlight, including the innovative integration of dual-EEG and frequency tagging techniques, as well as the relatively large sample size for hyperscanning research (25 dyads). However, some methodological and conceptual issues temper confidence in some of the authors' claims. Addressing the points outlined below would substantially strengthen the manuscript.

A. Method:

1.Participants: The manuscript does not report information on participants' musical training or rhythmic ability, which may represent a major confound for synchronization accuracy and sensitivity to changes in sound frequency (e.g., Scheurich et al., 2020). If these data were not collected, the authors should at least acknowledge this as a limitation and discuss the likely direction of its impact. Sample size justification should also be added to the manuscript.

2.Trial number: A critical limitation concerns the small number of trials in each condition. With only two trials per condition, the robustness of within-condition EEG estimates is likely reduced, despite the long trial duration (180 s each). This experimental choice requires justification. In addition, the authors should consider providing supplementary reliability analyses to increase confidence in the robustness of the data interpretation.

3.Another issue refers to the force-brightness mapping. The perceptual validity of the mapping between linear force and sound brightness is not demonstrated, leaving unclear whether "brightness" manipulations were perceived consistently across participants. More information is needed. For example, a pilot perceptual validation could demonstrate that participants reliably perceive increasing force as increased brightness.

4.I am also concerned with potential motor contamination of EEG signals. The finger force manipulation used to control sound brightness may introduce motor-related artifacts that could influence the EEG measures (see, for example, Timm et al., 2014). The authors should justify how motor contamination was addressed or mitigated.

5.The manuscript also lacks a description of hardware/software timing accuracy, timing alignment procedures, and drift correction - all essential components of hyperscanning methodology (e.g., see Zamm et al., 2024). Additional information on dual-EEG synchronization procedures is necessary.

B. Discussion:

6.In the discussion, certain claims extend beyond what the data directly support. For example, EEG-based source localization, particularly with a template head model, has limited anatomical precision, especially for hemispheric lateralization. The authors should temper these anatomical claims and discuss the limitations of template-based source localization more explicitly.

7.Interpretations involving participants' attentional expectations, "agency confusion," and hierarchical self-other processing along the auditory pathway should be presented more cautiously, as these mechanisms were not directly probed. Clearly distinguishing between empirically supported conclusions and more speculative theoretical interpretations would strengthen the discussion.

Cited references:

*Scheurich, R., Pfordresher, P. Q., & Palmer, C. Musical training enhances temporal adaptation of auditory-motor synchronization. Exp Brain Res 238, 81-92 (2020). https://doi.org/10.1007/s00221-019-05692-y

*Timm, J., SanMiguel, I., Keil, J., Schröger, E., & Schönwiesner, M. Motor intention determines sensory attenuation of brain responses to self-initiated sounds. J Cogn Neurosci 26(7), 1481-1489 (2014). https://doi.org/10.1162/jocn_a_00552

*Zamm, A., Loehr, J. D., Vesper, C., Konvalinka, I., Kappel, S. L., Heggli, O. A., Vuust, P., & Keller, P. E. A practical guide to EEG hyperscanning in joint action research: from motivation to implementation. Soc Cogn Affect Neurosci 19(1), nsae026 (2024). https://doi.org/10.1093/scan/nsae026

Reviewer #3: The study by Varlert et al. uses an auditory-motor synchronization paradigm to investigate how humans maintain a distinction between self- and other-generated sounds during joint action. The authors show that participants synchronize more effectively when their sounds are acoustically distinct and when leadership roles are assigned. I found the frequency-tagging approach to isolate neural responses to self- versus other-related stimuli particularly elegant, and the analyses and results are generally convincing. I have 3 major comments, as well as several smaller points, which I believe would strengthen the manuscript.

Major comments

Clarity of expression. The manuscript is very difficult to follow due to extremely long sentences and the use of technical terms before they are introduced or intuitively motivated. Early on, it is unclear what "inferring/coordinating from sound" refers to. The task description is especially hard to parse: even after repeated readings of the Fig. 1 caption, I struggled to understand (1) what the participant instructions actually were and (2) how exactly were the auditory signals experimentally manipulated. Several concepts appear abruptly (e.g., "brightness of sound", "frequency tagging"), though they are not widely familiar and require explanation for a general readership. In addition, some results are presented without even brief methodological context. Below, I point to a few particularly challenging passages; however, I would recommend that the manuscript be more broadly rewritten for clarity.

Contextualization and theoretical framing. In my opinion, the title (phrases like "self-other distinction from sound") strongly suggests a link to voice or speech processing, yet this literature is not addressed at all. Once it becomes clear that the study is not about voice or speech, the reader might then expect connections to other more typical auditory-motor coordination paradigms - such as tapping, joint rhythmic entrainment, or musical synchronization - but these are also largely neglected. Instead, the introduction moves rapidly to a very specific and technically complex paradigm without providing intuitive conceptual grounding. I recommend introducing the motivation more directly, clarifying what kind of "coordination from sound" the study targets (and why), and situating the work more clearly within both the vocal-agency and sensorimotor-synchronization literatures so that readers can understand how this paradigm extends or complements existing (more classical) approaches.

Results interpretation: top-down vs. bottom-up mechanisms. Several paragraphs in the introduction talk about hierarchical processes that might contribute to self-other distinction, but the argument remains difficult to follow, and the connection between these proposed top-down mechanisms and the present paradigm is not clearly articulated. For example, after the brief mention of "anticipation, adaptation, and ongoing planning" I was expecting that such processes would be manipulated or discussed, yet the manuscript never returns to them. In contrast, the broader auditory self-other literature often targets specific processes possibly guiding the hierarchical predictions: efference-copy mechanisms in self-voice monitoring (e.g., Whitford et al., 2019, Biological Psychiatry: CNN), memory-based self-voice representations (e.g., Iannotti et al., 2021, Cerebral Cortex), attentional and emotional biases (e.g., Pinheiro et al., 2022, Cortex), and even biases from concurrent sensory stimuli such as bone conduction (e.g., Orepic et al., 2023, RSOS). These lines of research provide a clearer theoretical grounding for understanding how self-related auditory information is processed. Moreover, many of self-other studies also converge on right-hemisphere specialization for self-related processing - both the sense of self more broadly (Northoff et al., 2006, NeuroImage) but also within auditory self-representation (e.g., Candini et al., 2018, NeuroImage: Clinical; Iannotti et al., 2021, Cerebral Cortex; Kaplan et al., 2008, SCAN). I therefore recommend that the authors relate their own right-hemisphere findings to this wider self-other literature, both in the Introduction (for context) and more importantly in the Discussion.

Minor

Line numbers would be helpful for the review

Title & abstract

I find "action coordination through sound" quite vague

The first part of abstract is also rather unclear. It is hard to understand the scope of the study: are people speaking in synchrony or tapping in rhythm, or something else? I am also not convinced by the statement "such interaction is challenging in real-life". I'd argue that the brain does do this quite well in real life.

Other vague expressions: "leader-follower roles", "stronger facilitation", "modality-specific" (which modalities other than auditory are manipulated?)

Introduction

Page 4:

"impossible in principle" - exclusive and too strong, not tested explicitly

"growing evidence point to a complementary and necessary involvement of selective attentional and predictive mechanisms (…)" - such as? References?

"Such top-down brain mechanisms (…)" - my major comments

Page 5:

"Here we combined dual-EEG and frequency-tagging techniques in a dyadic improvisation synchronization" - terms not clear for general readership

"an auditory-motor analogue of the mirror game paradigm" - please briefly mention what is the paradigm. The reader does not want to open another paper to properly understand this one

"Illustration of the improvisation synchronisation task in which (…)" and "This frequency tagging of the sound envelope enabled neural processing (…)" - long sentences very hard to follow

Fig 1: What instructions were given to participants? Upper panel - I find that the 2 rows are rather confusing than helping the interpretation - the only difference is the color, but then the color is introduced only below. The brightness aspect is not clear and not obvious if it is (and even should be) the same in both rows. Is this auditory manipulation evolving in time (the arrow)? Is this happening at each trial? Very hard to understand the figure without reading the methods.

"improvisation" suggests freedom, but "synchronisation" implies alignment. Not obvious how they are merged together in the paradigm.

The manuscript states that participants produced "original and synchronized fluctuations in brightness", but does not explain what these fluctuations should look like, whether they followed an external cue, or how participants knew they were on time with one another.

The manipulation of brightness through finger pressure is highly unconventional and is not sufficiently explained in intuitive terms.

Why 33 and 47 Hz exactly?

Isn't it critical to mention that these amplitude fluctuations cannot be consciously perceived?

Intermodulation frequencies - not obvious why they would indicate integration. Does their processing necessarily reflects integration? Could low-level auditory processing be really decoupled from higher-level mechanisms (e.g. paying attention to self/other identity)?

Results

Coherence - briefly mention how it is computed

ANOVAs - was there an interaction term and why yes or why not (in all analyses)?

Fig 2 - "1 × 95% CI of the mean computed for within-subject designs" - not clear

Why up to 5 Hz?

Page 9: Why is Leadership variable now modeled differently (3 levels instead of 2)? Why is this not consistent across analyses (or both reported for all)?

Fig 3 - it would be helpful to indicate what is significant. The same for some other plots.

Why averaging across all EEG channels?

Fig 4 - What is the take-home message of this plot? Left: There are a few other prominent peaks than the 4 indicated ones. What could they represent? Is it then justified to only use 14 and 80 for further analyses? Can there be other markers of integration (e.g. some other nonlinear combination of 33 and 47)? Right: The 80 peak does not seem to stand out from many others. Can it therefore be used reliably in further analyses?

"suggesting an increase in the neural tracking (…) not only originating from peripheral changes in sound brightness but also centrally from selective enhancement at the brain level" Do we even know that there are any peripheral changes (I assume here you mean the cochlea or auditory nerve?) Not sure this statement is justified if this was not manipulated.

Fig 5 - what are the units on the x and y axes?

"These analyses also show that it is the right hemisphere that benefits the most from designated leadership roles, which could reflect the spectral superiority of this hemisphere" What is "spectral superiority of a brain hemisphere"?

Fig 6 - Could the response be modulated by the absolute value of the 2 chosen frequencies? Why the choice of 200 and 500?

What about the effect of Leadership in the "Self-Other neural integration/segregation" part? Do you observe similar effects for other prominent peaks from Fig 4?

The dual-EEG setup is interesting, but only within-participant analyses are reported. Have the authors explored inter-brain synchrony or leader-follower dynamics? Measures such as cross-brain coherence or correlation of frequency-tagged responses could reveal how neural coordination supports behavioral synchrony. Such findings would largely strengthen the manuscript.

Discussion

"Our work (…) demonstrates that the processes underlying interpersonal coordination are modality-specific." - How does this study show that? What modality other than auditory is tested?

Broader scope - why this work is important - could be strengthened. Specifically, there is a large body of literature demonstrating that auditory self-other confusion is related to auditory-verbal hallucinations, both in schizophrenia and in healthy individuals (e.g. Duggirala et al., 2024, Schizophrenia Bulletin; Waters et al., 2012, Schizophrenia Bulletin) and in other clinical changes in the sense of self (e.g. Voruz et al., 2024, Heliyon; Candini et al., 2018, NeuroImage: Clinical)

Methods

Sill not clear to me what exactly the instruction for the participants were. How was brightness of sound explained? How exactly is one told to lead?

# Author comment: Author response to Decision Letter 2

**DOI:** 10.1371/journal.pbio.3003935.r003

28 May 2026

Attachment Submitted filename: Responses_to_reviewers_R1.docx

# Peer review: Decision Letter 2

**DOI:** 10.1371/journal.pbio.3003935.r004

**Authors:** Christian Schnell, PhD

26 Jun 2026

Dear Dr Varlet,

Thank you for your patience while we considered your revised manuscript "Mechanisms underpinning the self-other distinction during interpersonal auditory-motor coordination" for publication as a Research Article at PLOS Biology. This revised version of your manuscript has been evaluated by the PLOS Biology editors, the Academic Editor and two the original reviewers.

Based on the reviews and on our Academic Editor's assessment of your revision, we are likely to accept this manuscript for publication, provided you satisfactorily address the following data and other policy-related requests:

* We would like to suggest a different title to improve its accessibility for our broad audience:

Right-hemisphere-dominant neural mechanisms facilitate auditory self-other distinction and coordination in human dyads

* Please mention in the competing interests section that David Poeppel is a member of PLOS Biology's editorial board.

* DATA POLICY:

You may be aware of the PLOS Data Policy, which requires that all data be made available without restriction: http://journals.plos.org/plosbiology/s/data-availability. For more information, please also see this editorial: http://dx.doi.org/10.1371/journal.pbio.1001797

Note that we do not require all raw data. Rather, we ask that all individual quantitative observations that underlie the data summarized in the figures and results of your paper be made available in one of the following forms:

1) Supplementary files (e.g., excel). Please ensure that all data files are uploaded as 'Supporting Information' and are invariably referred to (in the manuscript, figure legends, and the Description field when uploading your files) using the following format verbatim: S1 Data, S2 Data, etc. Multiple panels of a single or even several figures can be included as multiple sheets in one excel file that is saved using exactly the following convention: S1_Data.xlsx (using an underscore).

2) Deposition in a publicly available repository. Please also provide the accession code or a reviewer link so that we may view your data before publication.

Regardless of the method selected, please ensure that you provide the individual numerical values that underlie the summary data displayed in the following figure panels as they are essential for readers to assess your analysis and to reproduce it: 2BD, 3, 6 and 8AB.

NOTE: the numerical data provided should include all replicates AND the way in which the plotted mean and errors were derived (it should not present only the mean/average values).

Please also ensure that figure legends in your manuscript include information on where the underlying data can be found, and ensure your supplemental data file/s has a legend.

Please ensure that your Data Statement in the submission system accurately describes where your data can be found.

* CODE POLICY

Per journal policy, if you have generated any custom code during the course of this investigation, please make it available without restrictions. Please ensure that the code is sufficiently well documented and reusable, and that your Data Statement in the Editorial Manager submission system accurately describes where your code can be found. More information on our Code Policy, what and how to share can be found here: https://journals.plos.org/plosbiology/s/code-availability

Please note that we cannot accept sole deposition of code in GitHub, as this could be changed after publication. However, you can archive this version of your publicly available GitHub code to Zenodo. Once you do this, it will generate a DOI number, which you will need to provide in the Data Accessibility Statement (you are welcome to also provide the GitHub access information). See the process for doing this here: https://docs.github.com/en/repositories/archiving-a-github-repository/referencing-and-citing-content

* Please move references, methodological details and supplementary results to the main manuscript file. We have no word count limit and want to make it as easy as possible for readers to find the information they are looking for.

As you address these items, please take this last chance to review your reference list to ensure that it is complete and correct. If you have cited papers that have been retracted, please include the rationale for doing so in the manuscript text, or remove these references and replace them with relevant current references. Any changes to the reference list should be mentioned in the cover letter that accompanies your revised manuscript.

In addition to these revisions, you may need to complete some formatting changes, which you will receive in a follow up email. A member of our team will be in touch with a set of requests shortly. If you do not receive a separate email within a few days, please assume that checks have been completed, and no additional changes are required.

We expect to receive your revised manuscript within two weeks.

To submit your revision, please go to https://www.editorialmanager.com/pbiology/ and log in as an Author. Click the link labelled 'Submissions Needing Revision' to find your submission record. Your revised submission must include the following:

- a cover letter that should detail your responses to any editorial requests, if applicable, and whether changes have been made to the reference list

- a Response to Reviewers file that provides a detailed response to the reviewers' comments (if applicable, if not applicable please do not delete your existing 'Response to Reviewers' file.)

- a track-changes file indicating any changes that you have made to the manuscript.

NOTE: If Supporting Information files are included with your article, note that these are not copyedited and will be published as they are submitted. Please ensure that these files are legible and of high quality (at least 300 dpi) in an easily accessible file format. For this reason, please be aware that any references listed in an SI file will not be indexed. For more information, see our Supporting Information guidelines:

https://journals.plos.org/plosbiology/s/supporting-information

*Published Peer Review History*

Please note that you may have the opportunity to make the peer review history publicly available. The record will include editor decision letters (with reviews) and your responses to reviewer comments. If eligible, we will contact you to opt in or out. Please see here for more details:

https://plos.org/published-peer-review-history/

*Press*

Should you, your institution's press office or the journal office choose to press release your paper, please ensure you have opted out of Early Article Posting on the submission form. We ask that you notify us as soon as possible if you or your institution is planning to press release the article.

*Protocols deposition*

To enhance the reproducibility of your results, we recommend that if applicable you deposit your laboratory protocols in protocols.io, where a protocol can be assigned its own identifier (DOI) such that it can be cited independently in the future. Additionally, PLOS ONE offers an option for publishing peer-reviewed Lab Protocol articles, which describe protocols hosted on protocols.io. Read more information on sharing protocols at https://plos.org/protocols?utm_medium=editorial-email&utm_source=authorletters&utm_campaign=protocols

Please do not hesitate to contact me should you have any questions.

Sincerely,

Christian

Christian Schnell, PhD

Senior Editor

cschnell@plos.org

PLOS Biology

------------------------------------------------------------------------

Reviewer remarks:

Reviewer #2: The authors have responded carefully and comprehensively to the issues raised in a previous version of the manuscript. The manuscript has been substantially improved in terms of clarity, methodological transparency, theoretical framing, and interpretation of the findings. While some issues (e.g., formal validation of the brightness manipulation and empirical assessment of reliability) remain only partially resolved, these limitations are now adequately acknowledged and do not undermine the main conclusions of the study. I therefore support publication of the manuscript in its revised form.

Reviewer #3: I thank the authors for addressing my comments and applaud them for such a thorough revision.

# Author comment: Author response to Decision Letter 3

**DOI:** 10.1371/journal.pbio.3003935.r005

6 Jul 2026

Attachment Submitted filename: Responses_to_reviewers_R1_auresp_3.docx

# Editor report: Decision Letter 3

**DOI:** 10.1371/journal.pbio.3003935.r006

**Authors:** Christian Schnell, PhD

13 Jul 2026

Dear Dr Varlet,

Thank you for your patience while we considered your revised manuscript "Lateralised neural mechanisms facilitate auditory self-other distinction and coordination in human dyads " for publication as a Research Article at PLOS Biology.

Thank you also for largely addressing the editorial requests. There is one point, however, which needs further changes.

The references in the supplementary information file need to be moved to the main manuscript files. The reason for this is that the literature databases do not process references from supplementary information files, so the authors of those references would not receive any credit for these citations. You can then use the reference numbers from the main manuscript file to cite the papers in the supplementary information file.

Everything else looked and we'll be able to formally accept the your paper for publication once this is sorted.

In addition to these revisions, you may need to complete some formatting changes, which you will receive in a follow up email. A member of our team will be in touch with a set of requests shortly. If you do not receive a separate email within a few days, please assume that checks have been completed, and no additional changes are required.

We expect to receive your revised manuscript within one week.

To submit your revision, please go to https://www.editorialmanager.com/pbiology/ and log in as an Author. Click the link labelled 'Submissions Needing Revision' to find your submission record. Your revised submission must include the following:

- a cover letter that should detail your responses to any editorial requests, if applicable, and whether changes have been made to the reference list

- a Response to Reviewers file that provides a detailed response to the reviewers' comments (if applicable, if not applicable please do not delete your existing 'Response to Reviewers' file.)

- a track-changes file indicating any changes that you have made to the manuscript.

NOTE: If Supporting Information files are included with your article, note that these are not copyedited and will be published as they are submitted. Please ensure that these files are legible and of high quality (at least 300 dpi) in an easily accessible file format. For this reason, please be aware that any references listed in an SI file will not be indexed. For more information, see our Supporting Information guidelines:

https://journals.plos.org/plosbiology/s/supporting-information

*Published Peer Review History*

Please note that you may have the opportunity to make the peer review history publicly available. The record will include editor decision letters (with reviews) and your responses to reviewer comments. If eligible, we will contact you to opt in or out. Please see here for more details:

https://plos.org/published-peer-review-history/

*Press*

Should you, your institution's press office or the journal office choose to press release your paper, please ensure you have opted out of Early Article Posting on the submission form. We ask that you notify us as soon as possible if you or your institution is planning to press release the article.

*Protocols deposition*

To enhance the reproducibility of your results, we recommend that if applicable you deposit your laboratory protocols in protocols.io, where a protocol can be assigned its own identifier (DOI) such that it can be cited independently in the future. Additionally, PLOS ONE offers an option for publishing peer-reviewed Lab Protocol articles, which describe protocols hosted on protocols.io. Read more information on sharing protocols at https://plos.org/protocols?utm_medium=editorial-email&utm_source=authorletters&utm_campaign=protocols

Please do not hesitate to contact me should you have any questions.

Sincerely,

Christian

Christian Schnell, PhD

Senior Editor

cschnell@plos.org

PLOS Biology

# Author comment: Author response to Decision Letter 4

**DOI:** 10.1371/journal.pbio.3003935.r007

21 Jul 2026

Attachment Submitted filename: Responses_to_reviewers_R1_auresp_4.docx

# Editor report: Decision Letter 4

**DOI:** 10.1371/journal.pbio.3003935.r008

**Authors:** Christian Schnell, PhD

22 Jul 2026

Dear Dr Varlet,

Thank you for the submission of your revised Research Article "Lateralised neural mechanisms facilitate auditory self-other distinction and coordination in human dyads " for publication in PLOS Biology. On behalf of my colleagues and the Academic Editor, Jennifer Bizley, I am pleased to say that we can in principle accept your manuscript for publication, provided you address any remaining formatting and reporting issues. These will be detailed in an email you should receive within 2-3 business days from our colleagues in the journal operations team; no action is required from you until then. Please note that we will not be able to formally accept your manuscript and schedule it for publication until you have completed any requested changes.

Please take a minute to log into Editorial Manager at http://www.editorialmanager.com/pbiology/, click the "Update My Information" link at the top of the page, and update your user information to ensure an efficient production process.

PRESS

We frequently collaborate with press offices. If your institution or institutions have a press office, please notify them about your upcoming paper at this point, to enable them to help maximize its impact. If the press office is planning to promote your findings, we would be grateful if they could coordinate with biologypress@plos.org. If you have previously opted in to the early version process, we ask that you notify us immediately of any press plans so that we may opt out on your behalf.

We also ask that you take this opportunity to read our Embargo Policy regarding the discussion, promotion and media coverage of work that is yet to be published by PLOS. As your manuscript is not yet published, it is bound by the conditions of our Embargo Policy. Please be aware that this policy is in place both to ensure that any press coverage of your article is fully substantiated and to provide a direct link between such coverage and the published work. For full details of our Embargo Policy, please visit http://www.plos.org/about/media-inquiries/embargo-policy/.

Thank you again for choosing PLOS Biology for publication and supporting Open Access publishing. We look forward to publishing your study.

Sincerely,

Christian

Christian Schnell, PhD

Senior Editor

PLOS Biology

cschnell@plos.org
